# The evolution of click chemistry: from classical reactions to next-generation platforms

**DOI:** 10.1039/d6ra08128b

**Published:** 2026-10-07

**Authors:** Seyed Mohammad Mahdi Dadfar, Seyed Mohammadali Dadfar

**Affiliations:** a Institute of Nanoscale and Biobased Materials, Faculty of Materials Science and Technology, TU Bergakademie Freiberg Gustav-Zeuner-Str. 3 09599 Freiberg Germany Mahdi.Dadfar@esm.tu-freiberg.de; b Institute for Experimental Molecular Imaging, RWTH Aachen University Hospital Forckenbeckstraße 55 52074 Aachen Germany; c Ardena Oss Kloosterstraat 9 5349 AB Oss The Netherlands

## Abstract

Click chemistry has transformed modern synthetic chemistry by providing efficient, selective, and modular strategies for constructing complex molecular structures. Since its introduction, the field has expanded from classical synthetic reactions to bioorthogonal and next-generation click platforms, broadening its applications across organic synthesis, polymer science, materials engineering, chemical biology, medicinal chemistry, and biomedicine. This review summarizes the evolution of click chemistry across three major generations. The first generation includes classical reactions such as copper-catalyzed azide–alkyne cycloaddition (CuAAC), thiol–ene, thiol–yne, thiol–ene Michael addition (TEMA), Diels–Alder (DA) cycloadditions, and nucleophilic ring-opening reactions. The second generation focuses on bioorthogonal transformations, including Strain-Promoted Alkyne–Azide Cycloaddition (SPAAC), Strain-Promoted Alkyne–Nitrone Cycloaddition (SPANC), and inverse electron-demand Diels–Alder (IEDDA) reactions, which enable selective chemical modification in living systems. The third generation encompasses emerging platforms such as sulfur(vi) fluoride exchange (SuFEx) and phosphorus(v) fluoride exchange (PFEx), which provide enhanced structural tunability, external control, and broader application potential. For each reaction class, the review discusses fundamental mechanisms, key advantages, limitations, and representative applications, highlighting how click chemistry has evolved from efficient bond formation toward increasingly precise chemical control in biological and materials environments.

## Abstract 2

Click chemistry has transformed modern synthetic chemistry by providing efficient, selective, and modular strategies for constructing complex molecular structures.

## Graphical abstract

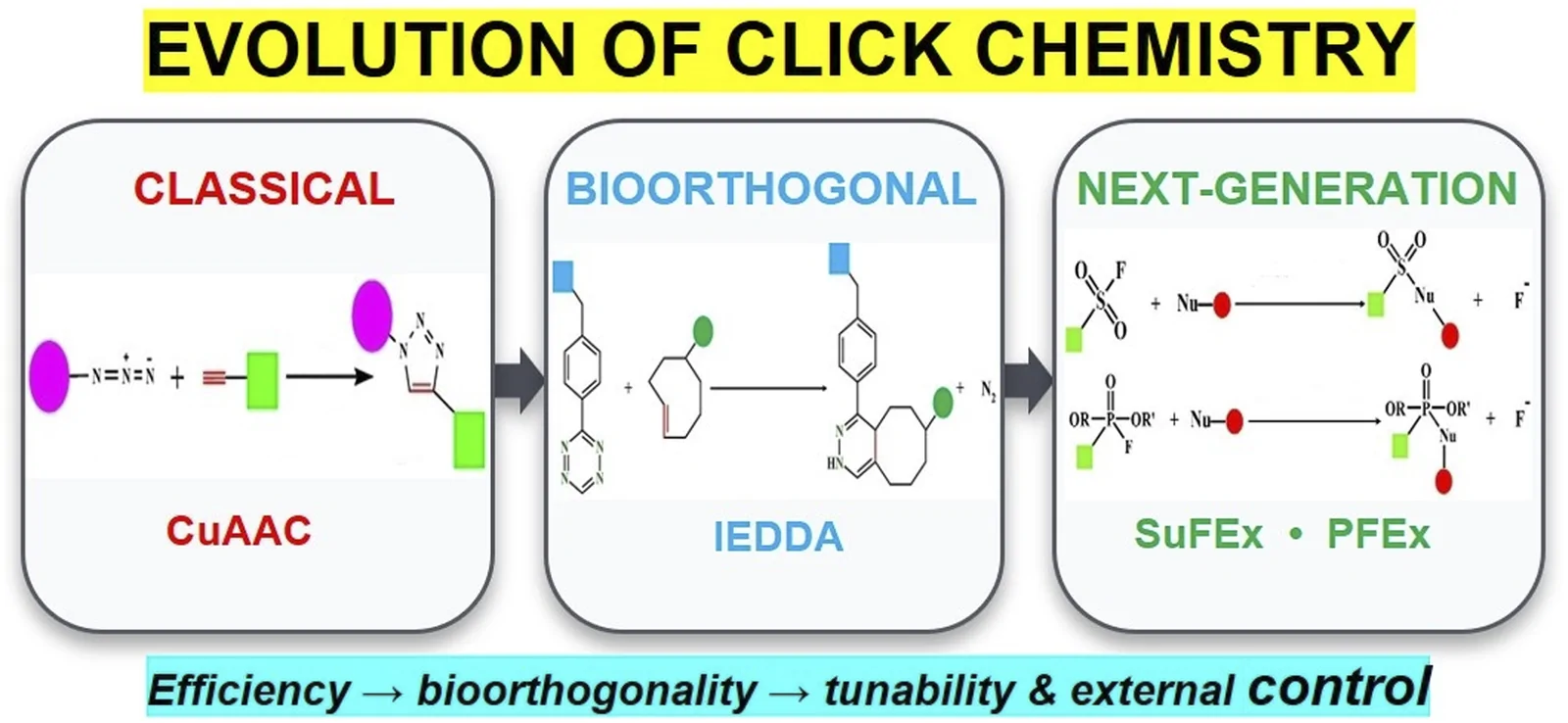

## 1 Introduction

Click chemistry was introduced by Sharpless and co-workers in 2001 as a conceptual framework for a class of highly efficient, modular, and selective reactions capable of joining molecular building blocks under simple reaction conditions.^1^ The goal was to provide robust synthetic transformations characterized by high yields, broad functional-group tolerance, stereospecificity, and minimal by-product formation. Shortly thereafter, independent studies by Meldal and by Rostovtsev, Fokin, and Sharpless established the CuAAC as the prototypical click reaction, providing a powerful and reliable route to 1,2,3-triazoles with exceptional regioselectivity and synthetic versatility.^2,3^

Since its introduction, the concept of click chemistry has expanded far beyond CuAAC to encompass a diverse range of transformations, including SPAAC,^4^ thiol–ene^5^ and thiol–yne^6^ reactions, DA cycloadditions, tetrazine ligation,^7^ and SuFEx chemistry.^8^ These reactions retain the fundamental characteristics of click chemistry while extending its applicability to polymer synthesis, materials engineering, surface modification, medicinal chemistry, nanotechnology, and chemical biology. A major milestone in the evolution of the field was the development of bioorthogonal click reactions by Bertozzi and co-workers, which enabled selective chemical transformations within living systems without interfering with native biological processes.^9,10^ This breakthrough established click chemistry as an indispensable tool for biomolecular labeling, molecular imaging, and therapeutic applications. The transformative impact of these discoveries was recognized by the 2022 Nobel Prize in Chemistry, awarded to Sharpless, Meldal, and Bertozzi.^11^

The widespread adoption of click chemistry is largely attributed to its unique combination of efficiency, selectivity, and operational simplicity.^12–14^ Click reactions typically proceed under mild conditions, often in aqueous media, with excellent chemoselectivity, high reaction yields, and minimal purification requirements. Furthermore, the ease of introducing clickable functional groups into small molecules, polymers, biomacromolecules, nanoparticles, and surfaces has enabled the rapid construction of increasingly complex molecular architectures. As a result, click chemistry has become a versatile platform for bioconjugation, drug delivery, molecular imaging, polymer functionalization, nanomaterial fabrication, and advanced materials design.^15–20^ Continuous advances in catalyst development, reaction engineering, bioorthogonal chemistry, SuFEx chemistry, photoclick reactions, and sustainable synthetic methodologies continue to expand both the scope and impact of click chemistry across multiple scientific disciplines.

Although click chemistry has been extensively reviewed, existing reviews have often emphasized individual reaction classes, specific application areas, or particular aspects such as bioorthogonal chemistry, polymer synthesis, or SuFEx.^6,9,18,21^ In contrast, this review provides an integrated analysis of the evolution of click chemistry by examining representative reactions within a common framework that connects their mechanisms, kinetics, operational requirements, advantages, limitations, and applications. A generation-based framework is adopted to trace the progression from classical click reactions to bioorthogonal ligations and, more recently, to externally controlled, orthogonal, and functionally tunable platforms. The review further compares the strengths and limitations of representative click reactions and examines their applications in chemical biology, drug discovery, nanomedicine, diagnostics, radiochemistry, polymer science, materials engineering, and surface modification. By integrating mechanistic analysis with application-oriented evaluation, this work provides a practical framework for understanding the selection and future development of click reactions. Current challenges, emerging trends, and future opportunities are also discussed, with the aim of identifying directions toward faster, more selective, sustainable, biocompatible, and externally controllable ligation strategies. The three-generation framework adopted in this review is intended as a conceptual classification of the evolving design principles of click chemistry rather than as a universally established historical taxonomy.

## 2 Evolution of click chemistry

### 2.1. First generation: classical click reactions

Classical click reactions comprise a group of highly efficient covalent bond-forming reactions that exhibit excellent chemoselectivity, high yields, broad functional-group tolerance, and operational simplicity under relatively mild reaction conditions.^1,14^ These reactions typically proceed with minimal by-product formation and generate stable linkages, making them particularly attractive for the rapid construction of complex molecular architectures.^1^ In view of their robustness and reliability, classical click reactions have found widespread applications in organic synthesis, polymer chemistry, materials science, surface modification, and medicinal chemistry.^5,14^ Their ability to efficiently connect diverse molecular building blocks has established them as fundamental tools for the synthesis of functional molecules and advanced materials.^1,5^ The most prominent examples of classical click reactions include the CuAAC, thiol–ene and thiol–yne reactions, TEMA, DA cycloadditions, and nucleophilic ring-opening reactions of strained heterocycles.^1,5,14^

#### 2.1.1. CuAAC

CuAAC (Fig. 1) is widely regarded as the archetypal click reaction and remains one of the most important transformations in modern synthetic chemistry and chemical biology. Prior to its development, the uncatalyzed Huisgen 1,3-dipolar cycloaddition between azides and alkynes required elevated temperatures, proceeded relatively slowly, and typically produced mixtures of 1,4- and 1,5-disubstituted 1,2,3-triazole regioisomers.^22^ A major breakthrough occurred in 2002 when Meldal^3^ and, independently, Rostovtsev, Fokin, and Sharpless^2^ demonstrated that the introduction of a Cu(i) catalyst dramatically altered the reaction pathway, resulting in a rate enhancement of up to 10^7^-fold and the highly selective formation of 1,4-disubstituted 1,2,3-triazoles under mild reaction conditions.^2,3,23,24^ The generally accepted CuAAC mechanism begins with coordination of Cu(i) to the terminal alkyne, followed by formation of a copper acetylide intermediate. Subsequent coordination of the organic azide generates a copper-containing six-membered metallacyclic intermediate, which undergoes cyclization to form a copper triazolide species. Final protonation releases the 1,4-disubstituted 1,2,3-triazole product and regenerates the Cu(i) catalyst (Fig. 2).^18,24,25^ CuAAC reactions are commonly catalyzed by Cu(i) salts such as CuI, CuBr, and Cu (CH_3_CN)_4_PF_6_, or by *in situ* reduction of Cu(ii) precursors (*e.g.*, CuSO_4_) using reducing agents such as sodium ascorbate.^26^ The synthetic utility of CuAAC derives from its high regioselectivity, broad functional-group tolerance, operational simplicity, and generally high conversion under mild conditions. The reaction can be performed in both protic and aprotic solvents, including aqueous media, and typically requires little or no protecting-group manipulation. Moreover, formation of a chemically, thermally, and metabolically stable 1,2,3-triazole linkage, together with minimal by-product generation, facilitates product isolation and contributes to the robustness of CuAAC as a general bond-forming strategy.^27–30^ These characteristics have supported applications across medicinal chemistry, chemical biology, polymer science, materials engineering, nanotechnology, and radiochemistry, including drug discovery, bioconjugation, biomolecular labeling, polymer functionalization, surface modification, nanomaterial fabrication, molecular imaging, and targeted drug delivery.^18,26,31–33^

**Fig. 1:**
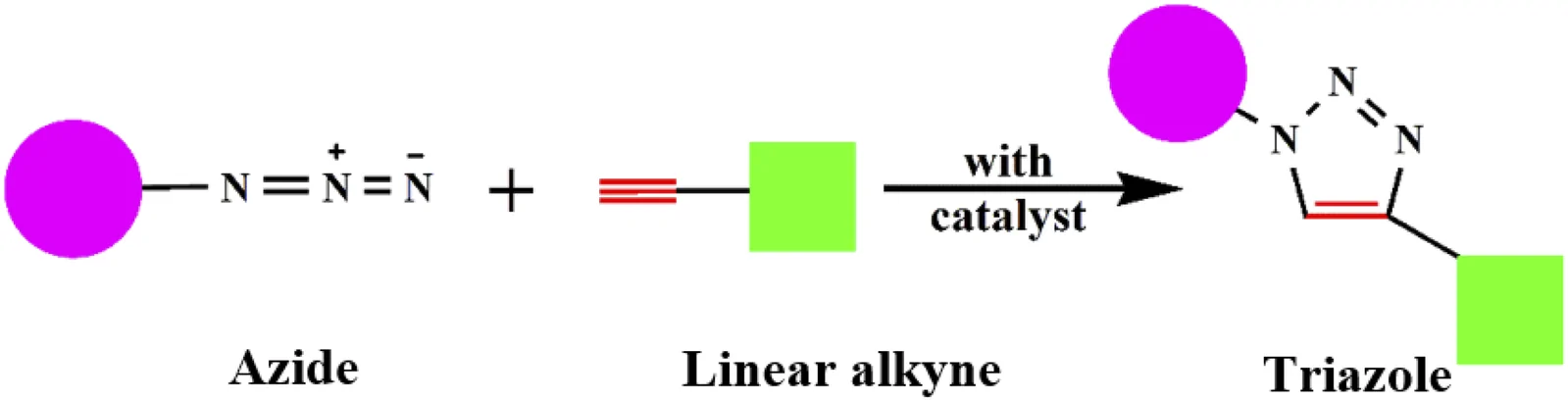
General reaction scheme for the CuAAC click reaction, adapted under the terms of the CC-BY license, ref. 25.

**Fig. 2:**
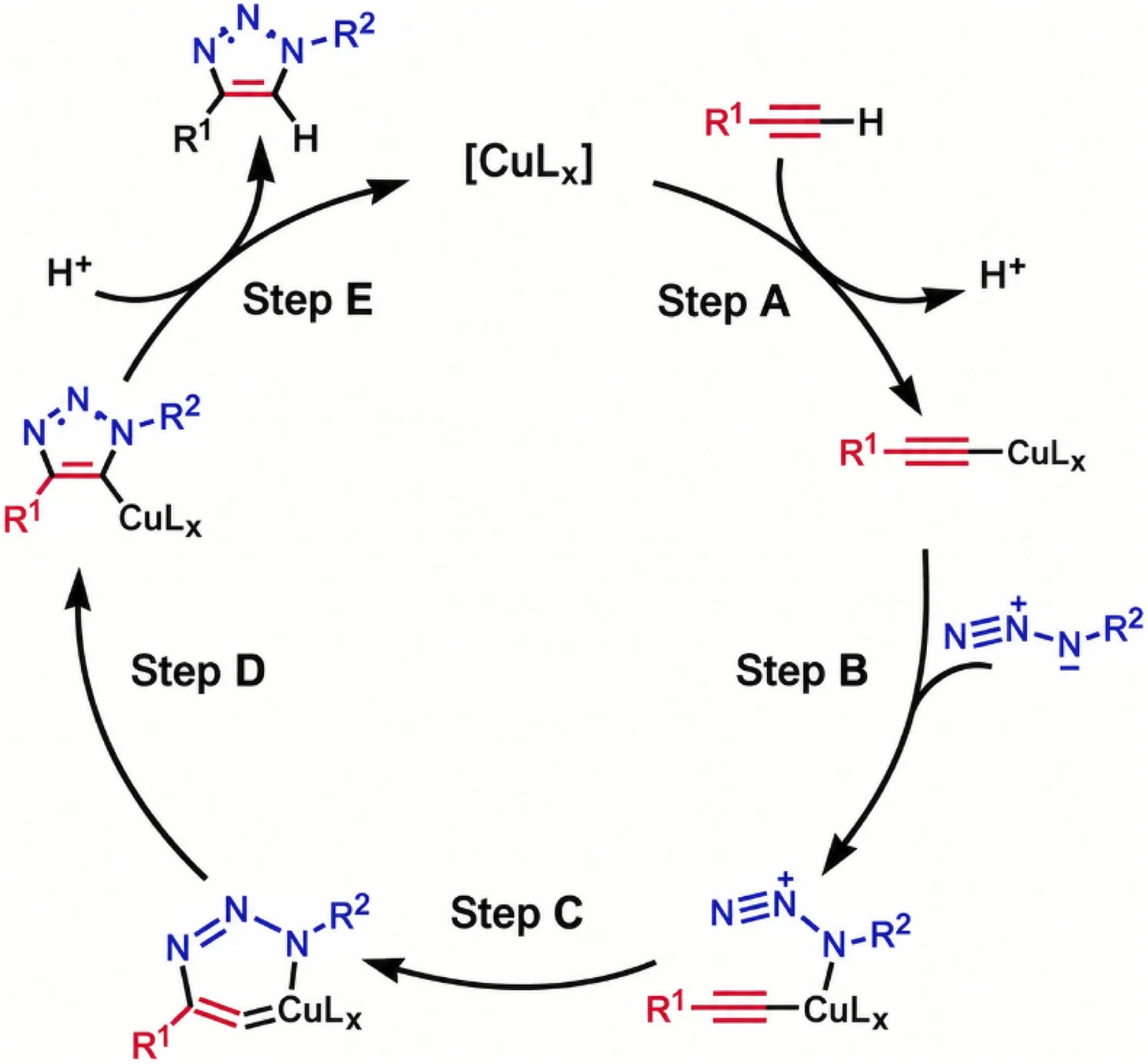
Proposed catalytic cycle of CuAAC leading to the formation of 1,4-disubstituted 1,2,3-triazoles. Adapted with permission from the Royal Society of Chemistry, copyright 2011, ref. 18.

The principal constraint of CuAAC in biological applications is its dependence on copper catalysis. Residual copper ions can induce cytotoxicity, oxidative stress, and degradation of sensitive biomolecules, thereby restricting direct application in living systems and creating an additional requirement for catalyst removal or sequestration.^34–38^

Several strategies have been developed to mitigate the limitations associated with copper catalysis. In particular, Cu(i)-stabilizing and ligand-accelerating systems employ appropriately designed ligands to increase the concentration and stability of catalytically active Cu(i) complexes, thereby substantially accelerating the cycloaddition and, in some cases, reducing the amount of copper required. However, these approaches remain copper-dependent and therefore do not completely eliminate concerns associated with metal exposure, cytotoxicity, or subsequent copper removal. Consequently, copper-free click reactions, particularly SPAAC, have been developed as important alternatives for applications in which metal-free conditions are required.^39,40^

#### 2.1.2. Thiol–ene

The thiol–ene reaction (Fig. 3), involving the hydrothiolation of carbon–carbon double bonds, has been known for more than a century. Initially developed as a synthetic transformation, it became widely employed in polymer and materials science for the preparation of highly uniform polymer networks and crosslinked films through the pioneering work of Hoyle, Bowman, and co-workers. More recently, the reaction has gained recognition as a click transformation because of its high efficiency, quantitative yields, orthogonality, and excellent tolerance toward a broad range of functional groups.^5,42,43^

**Fig. 3:**
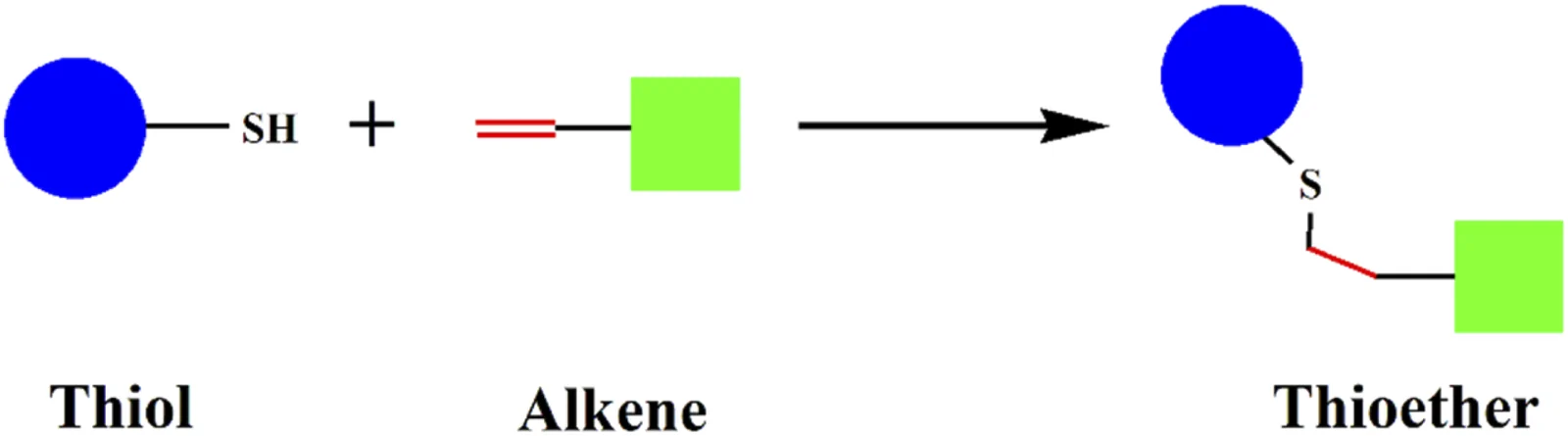
General reaction scheme for the thiol–ene click reaction, adapted under the terms of the CC-BY license, ref. 48.

The thiol–ene reaction typically proceeds through a free-radical step-growth mechanism initiated either thermally or photochemically through the generation of thiyl radicals using radical initiators such as 2,2-dimethoxy-2-phenylacetophenone (DMPA) or 2,2′-azobisisobutyronitrile (AIBN).^43^ Photoinitiation is most commonly employed due to its rapid reaction rates and precise spatiotemporal control. Under these conditions, thiyl radicals (RS˙) add across carbon–carbon double bonds to generate carbon-centered radical intermediates. These intermediates subsequently abstract a hydrogen atom from another thiol molecule, producing the desired thioether adduct while regenerating the thiyl radical and propagating the reaction chain (Fig. 4).^42–45^

**Fig. 4:**
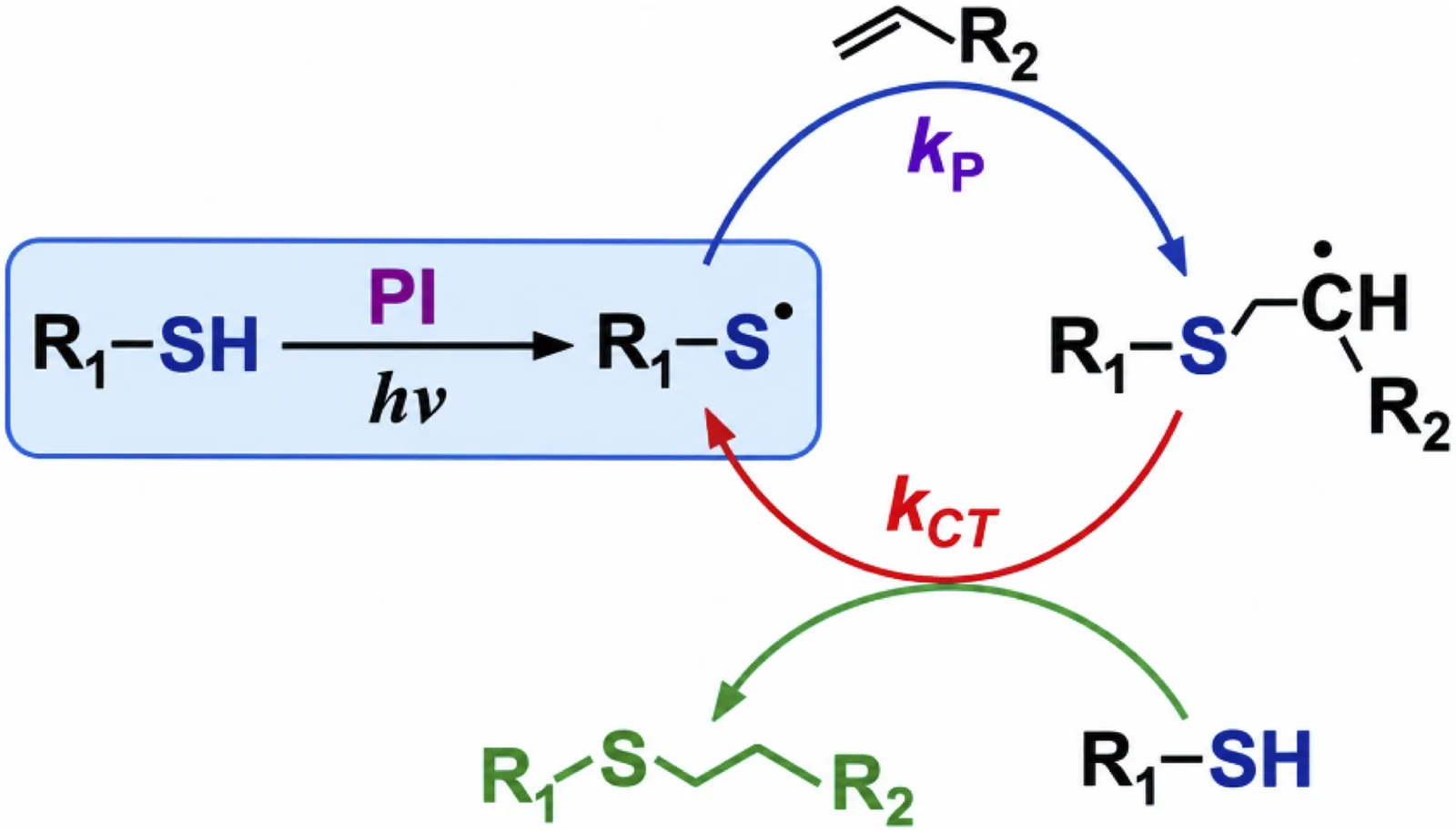
Proposed mechanism of the thiol–ene click reaction showing photoinitiation, radical propagation, and chain transfer, adapted under the terms of the CC-BY license, ref. 44.

Thiol–ene chemistry is distinguished by its rapid kinetics, high conversion, favorable atom economy, and broad tolerance toward chemically diverse functional groups.^5,42,43^ Its step-growth character is particularly advantageous for network formation because it promotes relatively uniform incorporation of multifunctional monomers and reduces network defects and shrinkage stresses, facilitating the preparation of well-defined polymer architectures.^42,43,46^ The ability to initiate the reaction thermally or photochemically further provides flexibility in processing and, in the latter case, enables spatial and temporal control over bond formation.

Several practical factors can influence the performance of thiol–ene reactions. Although substantially less sensitive to oxygen than conventional radical polymerizations, oxygen can still retard conversion under highly oxygen-rich conditions.^5,43^ The strong odor associated with many low-molecular-weight thiols can complicate handling and large-scale processing.^5^ In addition, sterically hindered or intrinsically unreactive alkenes may require longer irradiation times, higher initiator concentrations, or more energetic reaction conditions to reach high conversion.^42,43^ The radical nature of the process may also restrict its use with substrates that are particularly susceptible to radical-mediated side reactions.^5,42,43^

The combination of efficient step-growth polymerization and photochemical processability has made thiol–ene chemistry particularly valuable in polymer and materials research. Representative applications include polymer crosslinking, hydrogel fabrication, surface modification, photolithography, and additive manufacturing.^42,43,46^ Its relatively mild conditions and compatibility with biomolecular substrates have also enabled biomolecule conjugation, tissue engineering, drug-delivery systems, microfluidic devices, and stimuli-responsive materials.^5,42,43,45–47^ Thus, thiol–ene chemistry occupies an important position at the interface of polymer synthesis, photofabrication, and biomaterials engineering.

Compared with CuAAC, thiol–ene chemistry eliminates the need for metal catalysts and offers excellent temporal control under photochemical initiation, although its efficiency remains dependent on radical generation and alkene reactivity.

#### 2.1.3. TEMA

TEMA is a metal-free click transformation based on the nucleophilic conjugate addition of thiols to activated electron-deficient alkenes, such as acrylates, acrylonitriles, vinyl sulfones, and maleimides (Fig. 5). Unlike radical-mediated thiol–ene reactions, TEMA proceeds through a base- or nucleophile-catalyzed conjugate addition mechanism under mild conditions, eliminating the need for radical initiators or external irradiation.^5,6,49^ The reaction may follow either a base-initiated or a nucleophile-initiated pathway depending on the catalyst employed (Fig. 6). Although nucleophilic catalysis generally affords faster reaction rates and higher conversions than base catalysis, it may also lead to the formation of undesired side products.^50,51^ The reaction was first explored in organic synthesis through the classical Michael addition, but its potential as a click reaction was recognized following the work of Hoyle, Lowe, Bowman, and co-workers, who demonstrated its exceptional efficiency, orthogonality, and compatibility with polymer synthesis and bioconjugation.^5,6,49,52^ A defining feature of TEMA is its nucleophilic rather than radical reaction pathway, which largely eliminates oxygen inhibition and avoids the need for photoinitiators or radical initiators. Under appropriate catalytic conditions, the reaction provides rapid conversion, high chemoselectivity, and minimal by-product formation while retaining compatibility with multifunctional substrates.^5,6,49^ This combination of catalyst-controlled reactivity and mild processing conditions is particularly useful for applications requiring precise control over polymer network formation, hydrogel structure, and biomolecular conjugation.^5,47,52^ Compared with radical-mediated thiol–ene chemistry, TEMA offers superior chemoselectivity and oxygen tolerance under catalyst-controlled conditions, although its substrate scope is narrower than that of radical thiol–ene reactions.^5,43,53^ The scope of TEMA is nevertheless constrained by its dependence on activated Michael acceptors; unactivated alkenes generally display little or no reactivity.^6^ Unlike photoinitiated thiol–ene chemistry, the reaction begins as soon as the catalyst and reactants are combined, which limits temporal control over the onset of bond formation.^47^ Depending on the catalyst and reaction environment, competing processes such as disulfide formation, aza–Michael addition, ester hydrolysis, and transesterification can also become significant, particularly in multifunctional systems.^5,47,49^ In addition, the use of basic or nucleophilic catalysts may restrict compatibility with strongly acidic or pH-sensitive substrates.^6,49^

**Fig. 5:**
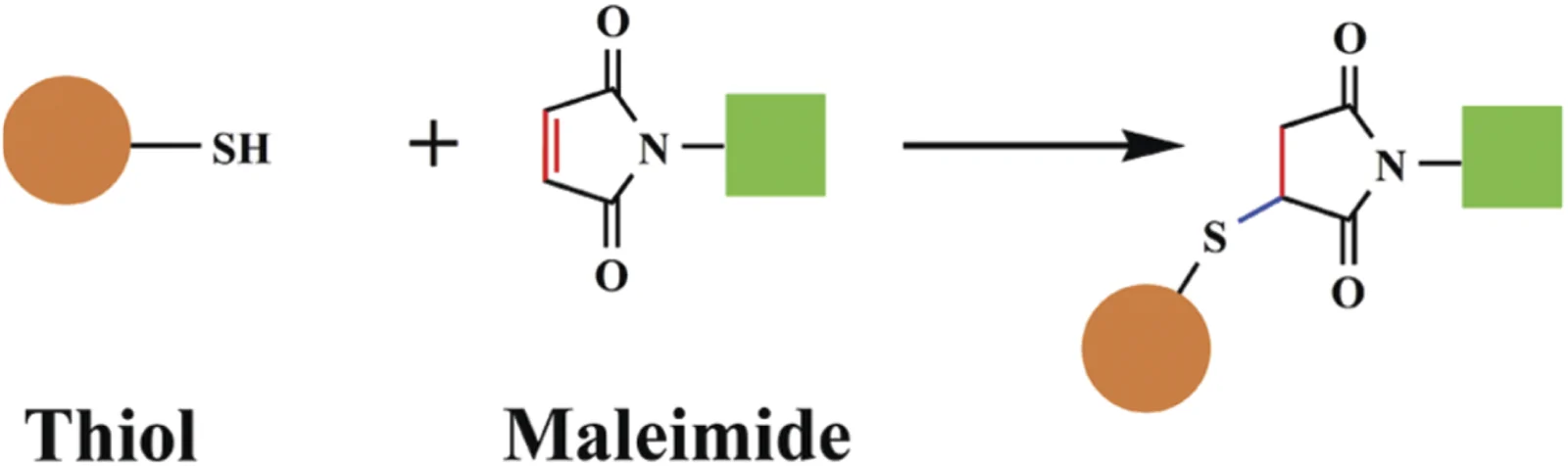
General reaction scheme for the thiol–ene click reaction, adapted under the terms of the CC-BY license, ref. 48.

**Fig. 6:**
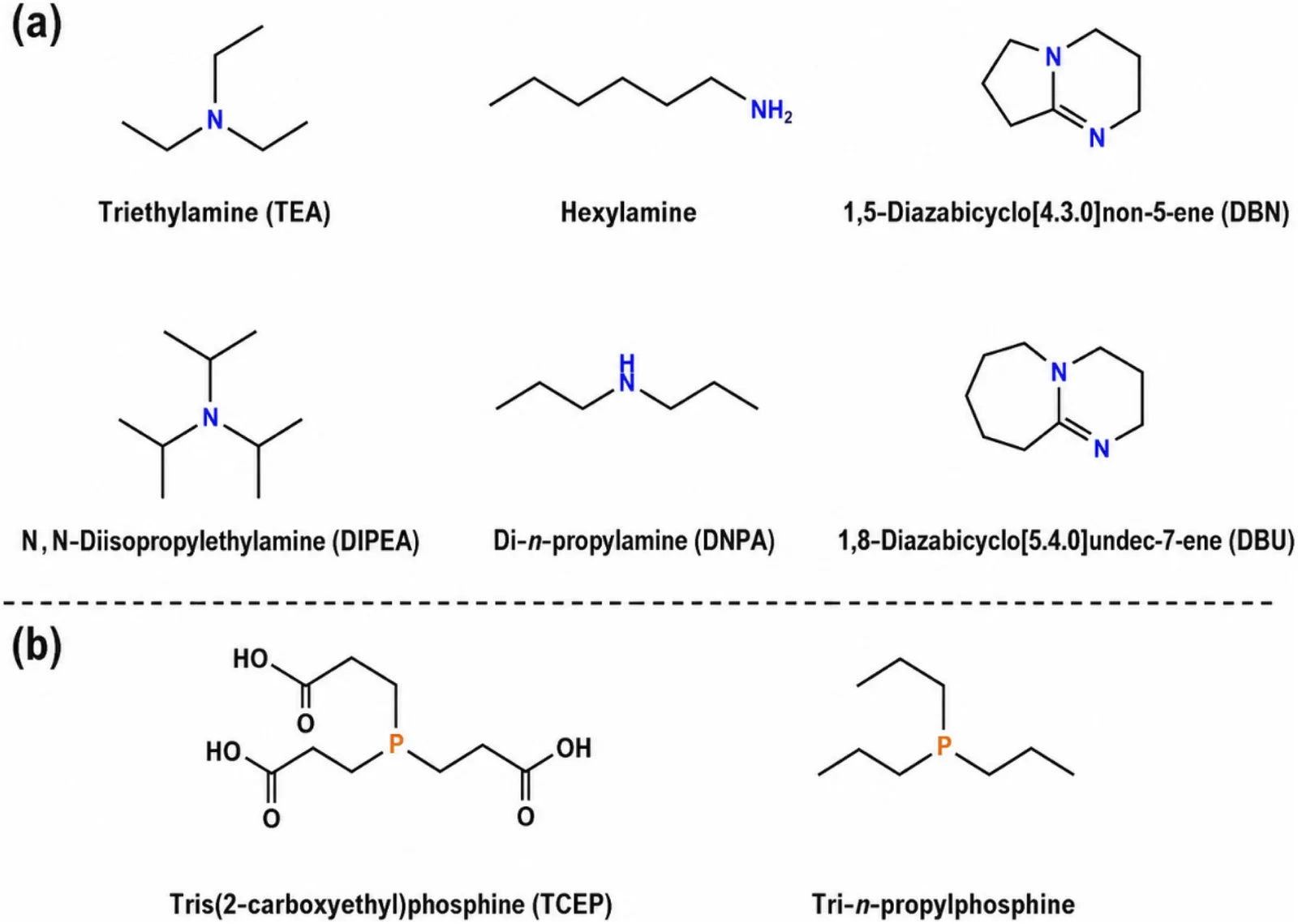
Examples of catalysts used in the (a) base-catalyzed and (b) nucleophile-catalyzed TEMA reaction, adapted under the terms of the CC-BY license, ref. 48.

TEMA has been exploited extensively in polymer functionalization, crosslinking, hydrogel fabrication, surface modification, bioconjugation, and drug-delivery systems.^5,6,49,52^ Its catalyst-controlled character has further enabled the preparation of sequence-controlled polymers, dynamic covalent materials, injectable biofunctional hydrogels, and advanced biomaterials for tissue engineering and biomedical applications.^6,43,49,52,54,55^ At the macromolecular level, TEMA has also been used to construct dendrimers, covalent molecular capsules, and functional network coatings, highlighting its utility for designing complex architectures with controlled connectivity.^6,43,54,55^

#### 2.1.4. Thiol–yne reactions (linear and cyclic alkynes)

Thiol–yne chemistry is a versatile member of the thiol-click family that involves the addition of thiols across carbon–carbon triple bonds (Fig. 7). Depending on the nature of the alkyne substrate, thiol–yne reactions can proceed either through radical-mediated addition to linear terminal or internal alkynes or through strain-promoted addition to cyclic alkynes. In radical thiol–yne reactions, thermal or photochemical initiation generates thiyl radicals that add across carbon–carbon triple bonds to produce vinyl sulfide intermediates. The initially formed vinyl sulfide subsequently undergoes a second radical thiol addition to afford the corresponding 1,2-dithioether product (Fig. 8). This stepwise addition provides exceptionally high atom economy and enables a greater degree of functionalization than thiol–ene chemistry, since each alkyne can react with two thiol molecules.^5,45,53^ In contrast, strained cycloalkynes such as cyclooctynes undergo spontaneous thiol addition driven by ring strain under catalyst-free conditions, making them attractive for bioconjugation and bioorthogonal applications.^9,34,56–58^ A key distinction between these two reaction pathways is the degree of thiol addition. In radical thiol–yne reactions involving linear alkynes, the initially formed vinyl sulfide intermediate remains reactive toward a second thiol addition, resulting in bis(thioether) products in which the original carbon–carbon triple bond is completely converted into a single bond. By comparison, reactions involving strained cycloalkynes generally terminate after a single addition event, yielding a vinyl sulfide in which the original triple bond is converted into a double bond while preserving the remaining unsaturation.^45,59–61^ Radical thiol–yne reactions have become widely employed because of their rapid kinetics, near-quantitative conversions, broad functional-group tolerance, and ability to introduce two thiol functionalities per alkyne, thereby enabling a higher functional-group density than conventional thiol–ene chemistry. Consequently, they are particularly well suited for the synthesis of highly crosslinked polymer networks, dendrimers, hyperbranched polymers, hydrogels, and multifunctional materials through efficient step-growth polymerization.^5,43,45,53^ Strain-promoted thiol–yne reactions further expand the scope of this chemistry by proceeding efficiently under mild, catalyst-free conditions, thereby facilitating applications in bioconjugation, surface modification, functional polymers, advanced coatings, and other biomaterials.^9,34^ The two thiol–yne variants present distinct practical challenges. Radical thiol–yne chemistry depends on radical initiation and can therefore be affected by oxygen inhibition and competing radical pathways. Selective monoaddition is also difficult because the initially generated vinyl sulfide can undergo a second thiol addition, favoring bis(thioether) formation unless the substrate and reaction conditions are carefully controlled.^5,43,45,53^ For strain-promoted systems, the principal constraints are the synthetic complexity and cost of strained cycloalkynes and the possibility of competing reactions with endogenous nucleophiles, particularly thiol-containing biomolecules, in biological environments.^9,34,56,61^ Given their distinct reactivity and exceptional conjugation efficiency, thiol–yne reactions serve as powerful platforms across polymer cross-linking, hydrogel synthesis, surface engineering, functional coatings, bioconjugation, targeted drug delivery, and the development of responsive nanomaterials and biomedical devices.^9,34,43,45,53,56,62^ Linear thiol–yne chemistry is particularly well suited for the synthesis of densely functionalized polymer networks, dendritic architectures, and surface-functionalized materials because each alkyne can accommodate two thiol additions. In contrast, strained cycloalkyne-based thiol–yne reactions are primarily employed for catalyst-free bioconjugation and other applications requiring rapid and selective C–S bond formation under mild or physiological conditions.^9,34,56,61^ Thus, whereas radical thiol–yne reactions are particularly valuable for the preparation of densely functionalized polymeric materials through sequential double addition, strain-promoted cycloalkyne reactions are better suited for catalyst-free conjugation under mild biological conditions.

**Fig. 7:**
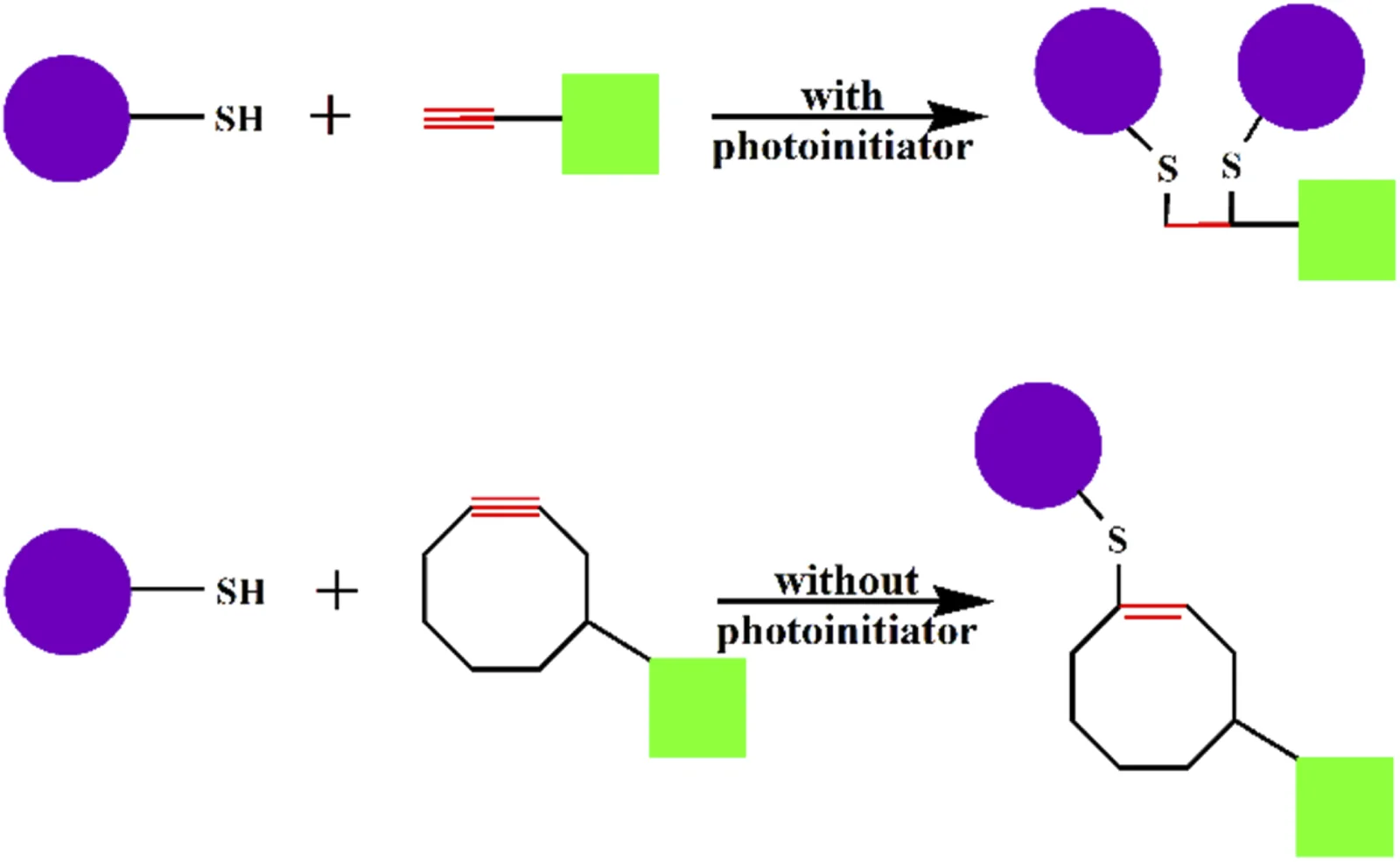
Reaction schemes for thiol–yne additions using linear alkynes (photo-initiated) *versus* strained cycloalkynes (photoinitiator-free), adapted under the terms of the CC-BY license, ref. 41.

**Fig. 8:**
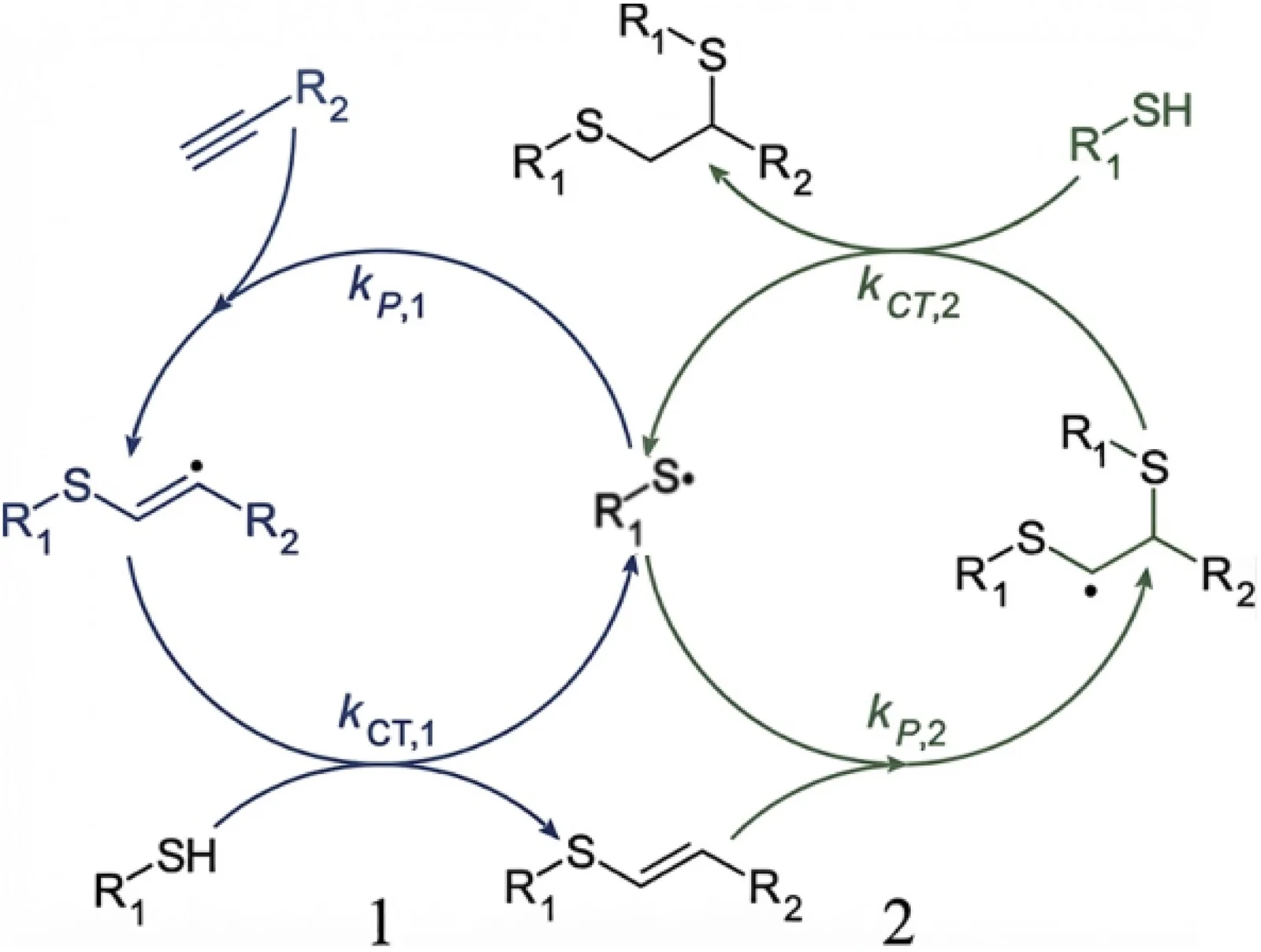
Reaction mechanism of the photo-initiated radical thiol–yne addition to a terminal linear alkyne, showing the sequential formation of the vinyl sulfide intermediate (1) and the final 1,2-dithioether adduct (2), adapted under the terms of the CC-BY license, ref. 45.

#### 2.1.5. DA cycloadditions

DA cycloaddition is one of the most powerful carbon–carbon bond-forming reactions in organic chemistry and has become an important member of the click chemistry toolbox because of its excellent atom economy, high regio- and stereoselectivity, and ability to proceed under mild, catalyst-free conditions.^63–65^ First reported by Otto Diels and Kurt Alder in 1928, the reaction involves a concerted, thermally allowed [4 + 2] cycloaddition between a conjugated diene and an electron-deficient dienophile to afford six-membered cyclic adducts without the formation of by-products.^66^ Owing to its efficiency, modularity, and broad substrate scope, the DA reaction has evolved into one of the most widely used click-like transformations in polymer chemistry, materials science, and chemical biology.^63–65^ Representative examples of classical DA cycloadditions are shown in Fig. 9,^48^ including reactions of heterocyclic dienes (*e.g.*, furan), acyclic conjugated dienes, and cyclic dienes with electron-deficient dienophiles such as maleimides, highlighting the versatility of this reaction family for constructing fused and bridged cyclic architectures. The reaction proceeds through a concerted pericyclic mechanism without requiring radical initiators or transition-metal catalysts and can be further accelerated by electron-rich dienes, electron-poor dienophiles, Lewis acid catalysis, or ring strain while maintaining excellent chemoselectivity and broad functional-group tolerance.^63–66^ The widespread use of DA chemistry arises from its excellent regio- and stereoselectivity, high to quantitative conversions, broad functional-group compatibility, exceptional atom economy, and operational simplicity. The reaction often proceeds under mild, catalyst-free conditions with minimal side-product formation, making it highly attractive for the synthesis of well-defined macromolecular architectures and functional materials.^63–65,67^ An additional feature that distinguishes DA chemistry from many irreversible click reactions is the availability of thermally reversible systems. Furan–maleimide adducts, for example, can undergo retro-Diels–Alder cleavage upon heating, providing a basis for dynamic covalent networks, self-healing polymers, recyclable materials, and stimuli-responsive systems.^63,64,67,68^ Despite these advantages, DA cycloadditions also present several limitations. Conventional DA reactions may require elevated temperatures or prolonged reaction times when less reactive dienes or dienophiles are employed, and their reaction rates are generally slower than those of CuAAC and IEDDA ligations.^63,65^ In addition, efficient cycloaddition often requires suitably activated reaction partners bearing electron-donating or electron-withdrawing substituents, while reaction efficiency and selectivity depend strongly on the electronic and steric properties of the diene and dienophile.^63–65^ Furthermore, the reversibility of certain DA adducts, although advantageous for dynamic and self-healing materials, may compromise thermal stability in applications requiring permanent covalent linkages.^63,64,68^ DA chemistry has found broad use in polymer synthesis, reversible crosslinking, hydrogel fabrication, surface functionalization, bioconjugation, drug-delivery systems, tissue engineering, and stimuli-responsive biomaterials.^63–65,68^ Its reversible variants are particularly valuable for adaptive materials in which covalent bonds must be repeatedly formed and cleaved, enabling self-healing, material reconfiguration, and recycling. More recently, these principles have been incorporated into advanced polymer networks, nanomaterials, and biomedical devices that exploit reversible covalent interactions for controlled structural adaptation.^63,64,68^

**Fig. 9:**
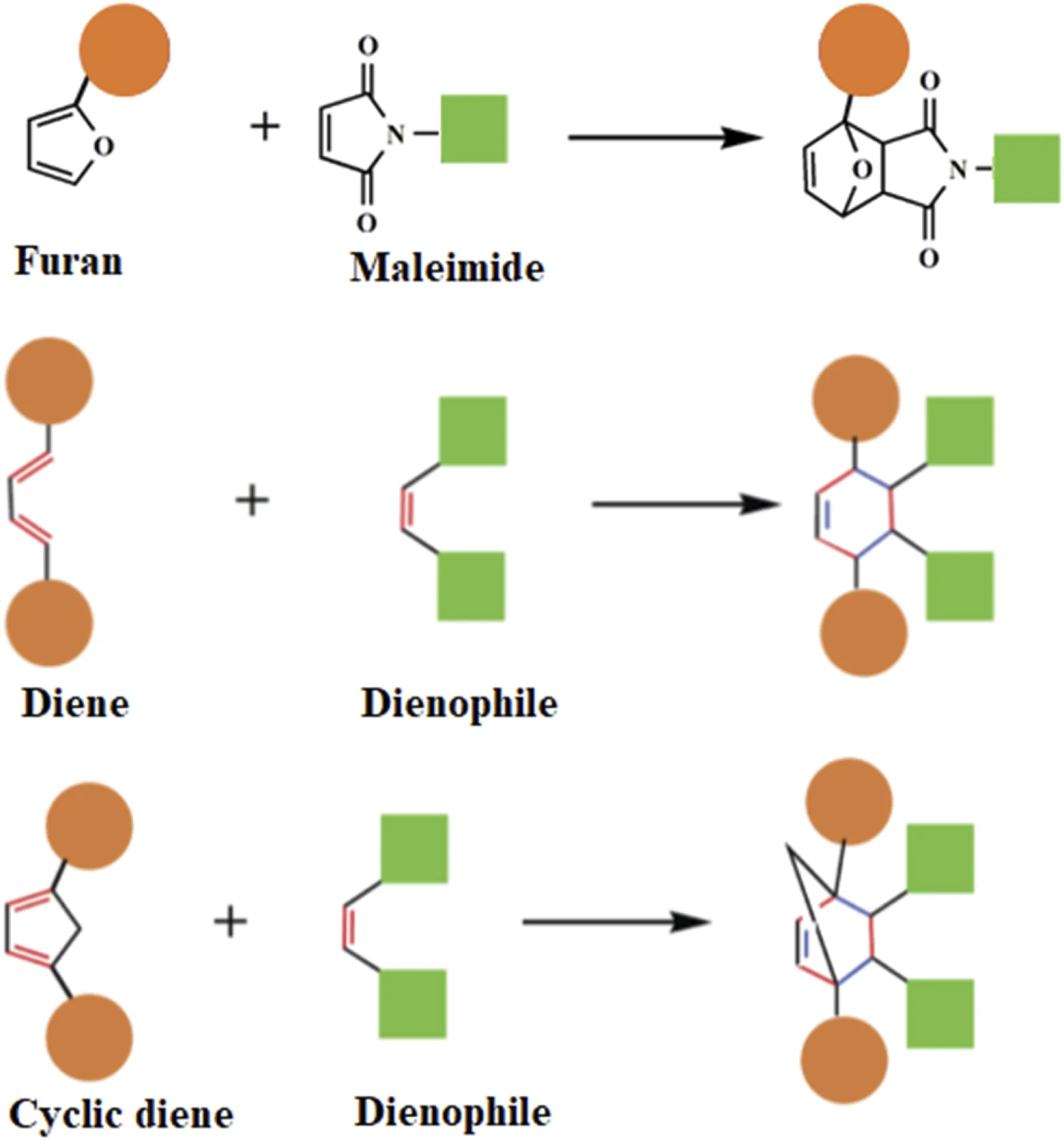
Representative examples of classical DA cycloadditions between dienes and electron-deficient dienophiles, adapted under the terms of the CC-BY license, ref. 48.

#### 2.1.6. Nucleophilic ring–opening reactions of epoxides

Nucleophilic ring-opening reactions of strained epoxides constitute one of the original classes of click reactions proposed by Sharpless and co-workers because of their high atom economy, modular bond-forming capability, and operational simplicity (Fig. 10).^69^ These transformations proceed through nucleophilic attack on the electrophilic carbon atom of the three-membered epoxide ring *via* an SN2-type mechanism, in which the substantial ring strain provides the thermodynamic driving force for efficient ring opening under relatively mild conditions, generally without significant by-product formation.^1,5,43,53,67^ Depending on the nucleophile employed, ring opening affords β-functionalized alcohols, including β-hydroxysulfides, β-amino alcohols, and β-azido alcohols through reactions with thiols, amines, and azides, respectively. In the thiol–epoxy pathway, base- or nucleophile-catalyzed deprotonation generates a reactive thiolate anion that attacks the less substituted carbon of the epoxide to produce β-hydroxysulfides with high efficiency and good regioselectivity.^5,43,70^ Primary and secondary amines react directly with epoxides to form β-amino alcohols and constitute the fundamental curing reaction in epoxy thermosets, coatings, and adhesives. However, because amines are relatively weak nucleophiles, homogeneous and heterogeneous catalysts are frequently employed to accelerate epoxide ring-opening reactions, although catalyst separation, recyclability, solvent use, and reaction conditions remain important practical considerations.^71–73^ In contrast, organic azides generally do not undergo direct nucleophilic ring-opening of epoxides under typical conditions. Instead, they are commonly transformed into amines *via* the phosphine-mediated Staudinger reaction, in which the azide reacts with a phosphine to form an iminophosphorane intermediate that subsequently hydrolyzes to the corresponding amine. The resulting amine can then participate in conventional epoxide ring-opening reactions.^74–79^ Because asymmetric epoxides can be attacked at either carbon atom, two regioisomeric products may be formed depending on the substrate structure and reaction conditions (Fig. 10).

**Fig. 10:**
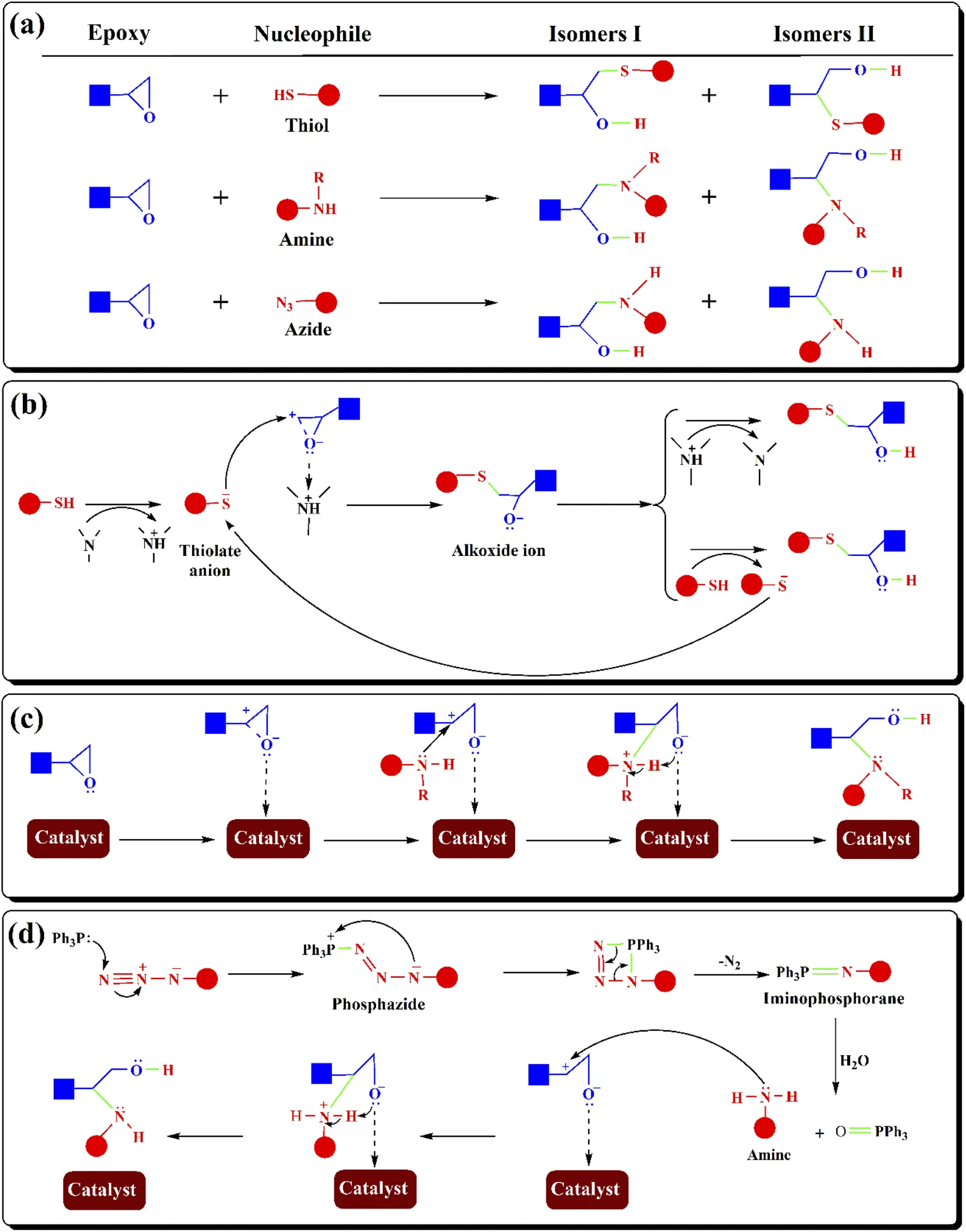
(a) The two possible regio-isomers expected from ring-opening reactions of epoxide by thiol, amine, and azide; proposed mechanisms for (b) thiol–epoxy reaction initiated by base catalyst, (c) amine–epoxy reaction over a heterogeneous catalyst, and (d) azide–epoxy reaction *via* Staudinger reaction, adapted under the terms of the CC-BY license, ref. 69.

The widespread use of nucleophilic epoxide ring-opening reactions arises from their high efficiency, excellent atom economy, operational simplicity, broad functional-group tolerance, and quantitative or near-quantitative conversions under relatively mild conditions. Unlike radical-mediated click reactions, these transformations proceed through ionic mechanisms and therefore do not require radical initiators, transition-metal catalysts, or external irradiation.^5,43,53,67,77,80^

Notwithstanding their strong utility, nucleophilic epoxide ring-opening reactions present notable drawbacks. In particular, reaction kinetics are highly sensitive to the steric and electronic profile of the epoxide, the specific nucleophile, the choice of catalyst, and the overall reaction conditions.^5,43,53,67^ Thiol–epoxy reactions generally require basic or nucleophilic catalysts to generate reactive thiolate species, whereas epoxy–amine systems often rely on catalytic activation because amines are generally less nucleophilic than thiolate species. Under strongly basic conditions, competing reactions such as epoxide homopolymerization, thiol oxidation, or multiple additions of multifunctional thiols and amines may occur, reducing network uniformity and product selectivity. Furthermore, the regioselectivity of epoxide ring opening is influenced by the steric and electronic characteristics of substituted epoxides, and asymmetric epoxides may yield mixtures of regioisomeric products. Careful control of catalyst type, catalyst loading, stoichiometry, and reaction conditions is therefore essential to minimize side reactions and achieve uniform polymer networks. Although azide-mediated epoxide functionalization provides valuable synthetic intermediates for subsequent click ligation, it generally involves additional reaction steps and is less commonly employed as a standalone click transformation than thiol–epoxy or epoxy–amine chemistry.^43,53,67^

Driven by high efficiency, operational simplicity, and broad functional-group tolerance, nucleophilic epoxide ring openings serve as vital methodologies in polymer chemistry, materials science, and biomedical engineering. These transformations have found widespread applications in polymer synthesis, epoxy resin curing, thermoset fabrication, hydrogel preparation, coatings, adhesives, nanocomposites, surface modification, post-polymerization functionalization, bioconjugation, and pharmaceutical synthesis.^5,43,53,67,69,74^ The β-functionalized alcohols generated during ring opening introduce hydroxyl groups that serve as versatile handles for subsequent crosslinking and chemical modification, thereby enabling the preparation of well-defined multifunctional materials. Among these reactions, thiol–epoxy chemistry has emerged as one of the most widely employed click transformations for the fabrication of highly crosslinked polymer networks and biomaterials because of its rapid kinetics, excellent orthogonality, and mild, metal-free reaction conditions.^81–86^ Epoxy–amine chemistry remains the cornerstone of industrial epoxy resin technologies, owing to its ability to produce thermosets with outstanding mechanical strength, thermal stability, and chemical resistance. Meanwhile, epoxide functionalization with azide-containing building blocks provides a versatile platform for the preparation of azide-functional polymers and small molecules that can undergo subsequent bioorthogonal ligation reactions, including CuAAC and SPAAC, thereby expanding the utility of epoxide chemistry in advanced functional materials and biomedical applications.^5,43,53,67,74^

Compared with many other classical click reactions, nucleophilic epoxide ring-opening reactions uniquely combine excellent atom economy, operational simplicity, and the simultaneous generation of hydroxyl functionalities, making them particularly attractive for polymer network formation, thermoset fabrication, and post-polymerization functionalization.

### 2.2. Second generation: bioorthogonal click chemistry

Bioorthogonal click chemistry represents the second major stage in the evolution of click chemistry, extending the original click chemistry concept to highly selective reactions that proceed within living systems without perturbing native biological and biochemical processes. Consequently, these reactions have become invaluable for the selective labeling and modification of biomolecules in cells and living organisms.^87–89^ Unlike the first generation of click reactions, many of which require metal catalysts or reaction conditions that are not fully compatible with living systems, bioorthogonal reactions are specifically designed to proceed rapidly under physiological conditions, in aqueous media, and at low reactant concentrations.^9,87,90^ To be considered truly bioorthogonal, a reaction should satisfy several stringent requirements. It must proceed rapidly under physiological conditions, even at low reactant concentrations; involve functional groups absent from native biological systems; generate products selectively and in high yield; and remain unaffected by water or the complex mixture of endogenous nucleophiles, electrophiles, oxidants, and reductants present in living cells and tissues.^87^

The development of bioorthogonal chemistry revolutionized chemical biology by allowing the selective labeling, imaging, tracking, and manipulation of biomolecules in real time without perturbing biological function.^91^ These reactions have become indispensable tools in glycobiology, proteomics, molecular imaging, drug delivery, diagnostics, and theranostics.^10^ Furthermore, advances in reaction kinetics and catalyst-free ligation strategies have enabled *in vivo* applications ranging from metabolic labeling and pretargeted imaging to targeted therapeutics and controlled drug release.^92^ Among the numerous bioorthogonal ligation strategies developed to date, the most widely employed include the Staudinger ligation, SPAAC, SPANC, IEDDA cycloaddition, and several other catalyst-free bioorthogonal ligation reactions.^9,10,90–93^

#### 2.2.1. SPAAC

SPAAC is a copper-free variant of azide–alkyne cycloaddition developed to overcome the cytotoxicity and limited biological compatibility associated with CuAAC. Introduced by Bertozzi and co-workers in 2004, SPAAC proceeds through a concerted [3 + 2] 1,3-dipolar cycloaddition between an organic azide and a strained cycloalkyne, typically a cyclooctyne derivative, under physiological conditions without the need for copper catalysts (Fig. 11).^4,9,34,38,41,56,94^ Mechanistically, the pre-distorted, high-energy C–C triple bond of the cyclooctyne undergoes thermally allowed cycloaddition with the azide to generate a stable 1,2,3-triazole linkage. The substantial ring strain of cyclooctynes, typically around 18 kcal mol^−1^, lowers the activation barrier and provides the driving force for spontaneous triazole formation while maintaining excellent atom economy and producing essentially no by-products.^9,34,56,95,96^ To enhance reaction kinetics, numerous cyclooctyne derivatives have been developed, including bicyclo[6.1.0]nonyne (BCN), dibenzocyclooctyne (DIBO), dibenzoazacyclooctyne (ADIBO/DIBAC/DBCO), biarylazacyclooctynone (BARAC), monofluorinated cyclooctyne (MOFO), and difluorinated cyclooctyne (DIFO), whose reactivity depends on their degree of ring strain and electronic activation (Fig. 12).^41^ Representative second-order rate constants (*k*_2_) for SPAAC vary by several orders of magnitude depending on the cyclooctyne structure and azide partner, with relatively lower reactivity reported for less activated cyclooctynes such as MOFO, and substantially faster reactions observed for activated systems such as DIFO, DIBO, DBCO, BARAC, and BCN. Representative values reported in the literature include approximately 10^−3^ to 10^−2^ M^−1^ s^−1^ for less reactive cyclooctynes and up to ∼1 M^−1^ s^−1^ or higher for highly activated derivatives. These values should, however, be interpreted with caution because SPAAC kinetics are strongly dependent on the structure of the azide, solvent, temperature, and experimental conditions. Thus, rather than representing a universal ranking, reported *k*_2_ values provide a useful indication of the relative reactivity of different cyclooctyne platforms. This tunability enables SPAAC reactivity to be adjusted for different biological and materials applications while requiring a balance between reaction rate, stability, solubility, steric properties, and synthetic accessibility.^12,97–103^

**Fig. 11:**
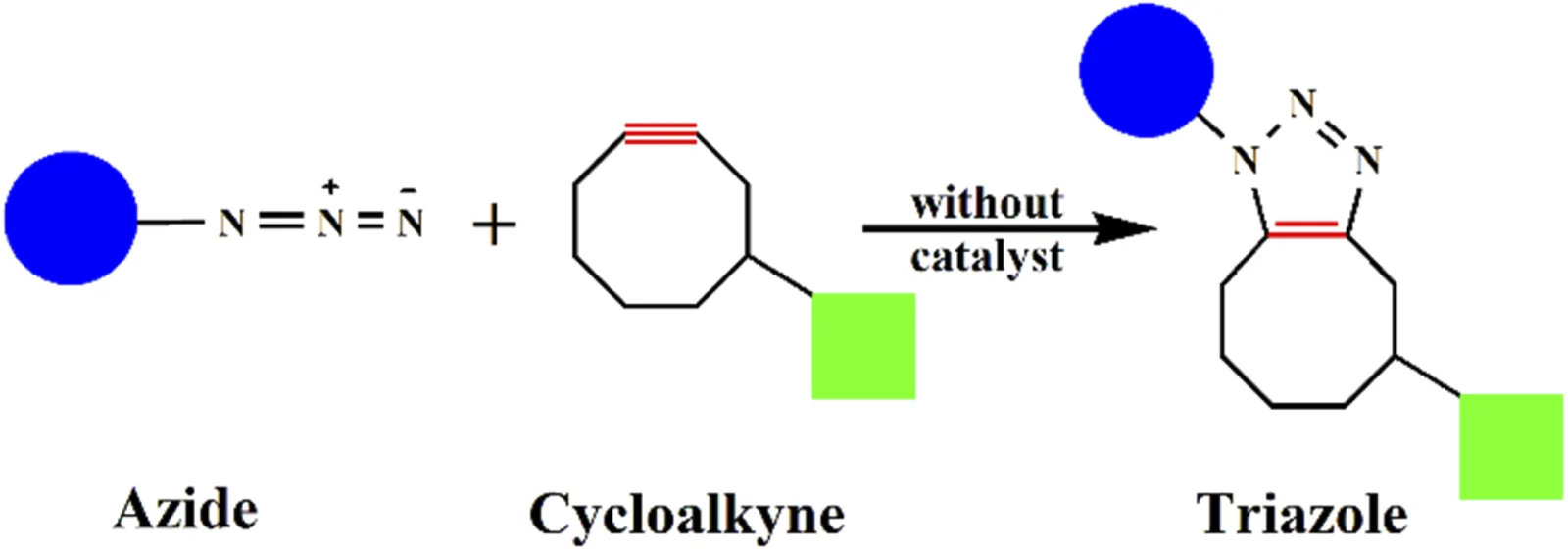
Schematic illustration of SPAAC, adapted under the terms of the CC-BY license, ref. 41.

**Fig. 12:**
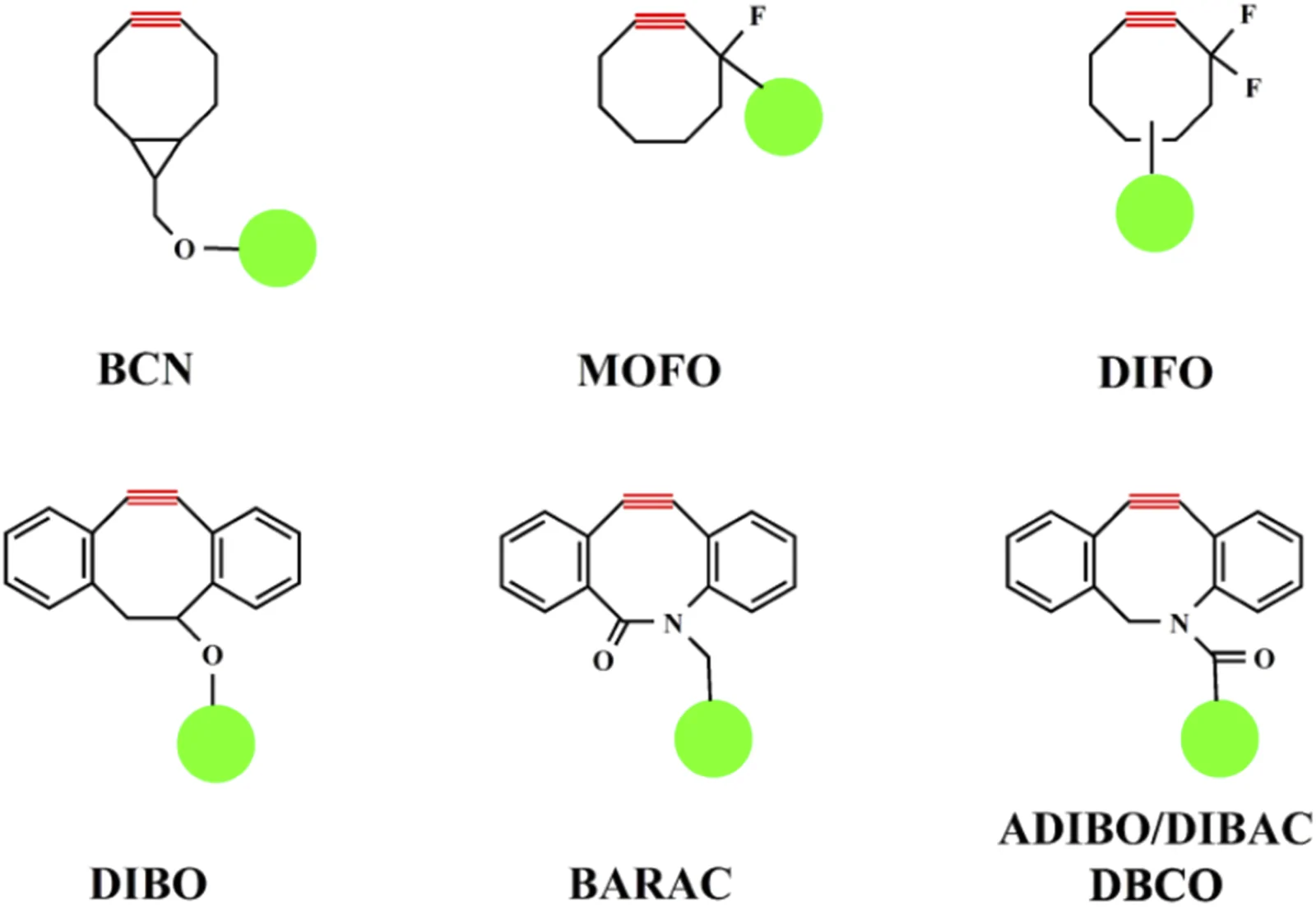
Some commonly used cyclooctynes for SPAAC, adapted under the terms of the CC-BY license, ref. 41.

The popularity of SPAAC stems from its outstanding chemoselectivity, metal-free nature, high tolerance for diverse functional groups, and excellent biocompatibility within living systems.^4,9,34,56–58,95,103,104^ Unlike CuAAC, SPAAC proceeds efficiently in aqueous media under physiological conditions without requiring cytotoxic copper catalysts or external reagents, making it particularly attractive for live-cell labeling, *in vivo* imaging, and biomolecule conjugation. The reaction exhibits outstanding bioorthogonality because both azides and strained cyclooctynes are essentially inert toward naturally occurring biological functional groups, enabling the selective conjugation of proteins, nucleic acids, carbohydrates, lipids, nanoparticles, and polymers while preserving their biological activity.^9,34,56,92,95,104–106^

Despite its excellent biological compatibility, SPAAC has several practical limitations. Its reaction kinetics are generally slower than those of IEDDA ligation and, in some cases, optimized CuAAC systems, which can necessitate relatively high reactant concentrations for rapid conjugation. Moreover, increasing cyclooctyne reactivity does not necessarily translate into improved overall performance, as highly activated cyclooctynes may exhibit greater synthetic complexity, reduced stability, increased hydrophobicity, or nonspecific interactions with biomolecules. The preparation of highly strained cyclooctynes is also synthetically demanding and comparatively costly, while their bulky and often hydrophobic structures can limit aqueous solubility or promote nonspecific interactions with biomolecules. In addition, classical SPAAC generally lacks strict regioselectivity and may generate mixtures of 1,4- and 1,5-disubstituted triazole products. Certain cyclooctyne derivatives may also undergo competing reactions with biological thiols or experience gradual degradation during storage, potentially affecting their stability and practical performance.^9,56,92,95,104,107^

The broad biological compatibility of SPAAC has established it as a versatile ligation strategy across chemical biology, biomedicine, and materials science. In chemical biology, it is widely used for protein, antibody, and oligonucleotide conjugation, metabolic labeling of glycans and lipids, nucleic-acid modification, and fluorescence-based imaging of biomolecules in living cells and organisms. Its catalyst-free nature has also enabled applications in pretargeted drug delivery, tissue engineering, and nanomedicine, where copper-mediated chemistry may be undesirable. Beyond biological applications, SPAAC has been employed for hydrogel crosslinking, surface functionalization, dendrimer and polymer synthesis, polymer–biomolecule conjugation, and the preparation of functional and stimuli-responsive nanostructures. Compared with CuAAC, SPAAC therefore offers a particularly valuable combination of catalyst-free operation, bioorthogonality, and compatibility with living systems, making it especially suitable for applications in which metal-free conjugation is essential.^4,9,33,34,56,92,95,96,104,108,109^ The principal distinctions between CuAAC and SPAAC are summarized in Table 1.

**Table 1:** Comparative overview of CuAAC and SPAAC click chemistries

Feature	CuAAC	SPAAC
Reaction type	Cu(i)-catalyzed 1,3-dipolar cycloaddition^1^	Copper-free strain-driven 1,3-dipolar cycloaddition^4,9,34,41,56^
Catalyst	Cu(i) catalyst required^1–3^	No catalyst required^4,34,56^
Driving force	Copper activation of terminal alkyne^2,3,26^	Release of cyclooctyne ring strain^4,9,34^
Alkyne substrate	Terminal alkyne^18,24^	Strained cyclooctyne (*e.g.*, BCN, DBCO, BARAC, DIFO)^12,41,97–103^
Reaction product	1,4-Disubstituted 1,2,3-triazole^2,3,23,24^	Mixture of 1,4- and 1,5-disubstituted 1,2,3-triazoles^4,34,107^
Reaction rate	Very fast^2,18,26^	Fast, but generally slower than CuAAC^9,34,56,104^
Living-cell applications	Limited unless copper toxicity is minimized^34–37^	Ideal for live-cell and *in vivo* applications^4,9,34,56,92,95,96,104^
Advantages	High yield, excellent regioselectivity, inexpensive alkynes, robust reaction, broad substrate scope^1,2,18,26^	Catalyst-free, highly biocompatible, excellent bioorthogonality, compatible with living systems^9,34,56,104^
Disadvantages	Copper toxicity, potential oxidative damage to biomolecules, catalyst removal required^18,37^	Slower kinetics, expensive strained cyclooctynes, bulky hydrophobic reagents, lower regioselectivity^34,56,95,104^
Typical applications	Polymer synthesis, dendrimers, surface modification, hydrogels, materials chemistry, bioconjugation^1,18,26^	Live-cell labeling, metabolic glycan labeling, protein conjugation, drug delivery, tissue engineering, nanomedicine, biomaterials^4,9,33,56,104,108^
Representative cycloalkyne/catalyst	CuSO_4_/sodium ascorbate, CuBr, TBTA ligand^26^	BCN, DBCO, DIBO, BARAC, DIFO^12,97–103^
Overall suitability	Best for synthetic chemistry and materials science^18,31,32^	Best for biological and biomedical applications^4,9,34,56,92,95,96,104^

#### 2.2.2. IEDDA cycloaddition

IEDDA cycloaddition is one of the fastest and most powerful bioorthogonal click reactions, involving a concerted [4 + 2] cycloaddition between an electron-deficient diene, most commonly a 1,2,4,5-tetrazine, and an electron-rich or strained dienophile, such as *trans*-cyclooctene (TCO), norbornene, cyclopropenes, or strained cyclooctynes (Fig. 13).^94,110,111^ In contrast to the classical DA reaction, IEDDA proceeds through an inverse frontier molecular orbital interaction in which the low-lying LUMO of the tetrazine reacts with the HOMO of the dienophile, rapidly forming a bicyclic intermediate that spontaneously extrudes molecular nitrogen (N_2_), yielding a stable dihydropyridazine or pyridazine product with excellent atom economy and irreversible bond formation.^7,92,93,111,112^

**Fig. 13:**
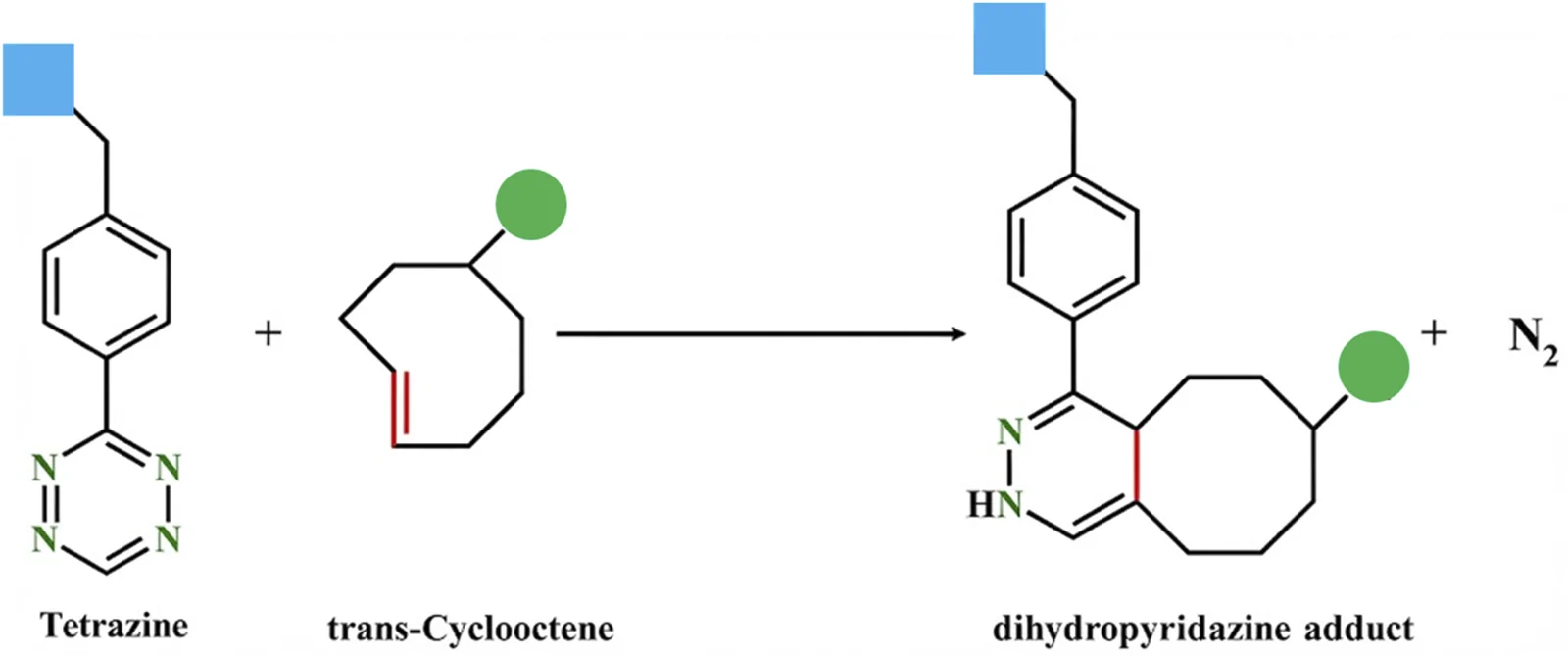
Representative IEDDA cycloaddition between a tetrazine and TCO.

Since its introduction into chemical biology by Fox and co-workers, IEDDA chemistry has become one of the most widely employed bioorthogonal ligation strategies because of its exceptionally fast kinetics, catalyst-free operation, and ability to proceed efficiently under physiological conditions without requiring metal catalysts, external reagents, or irradiation. Its remarkable success arises from its ultrafast reaction kinetics, outstanding chemoselectivity, quantitative conversions, catalyst-free operation, and excellent compatibility with aqueous media and biological systems.^7,92,93,111,113–116^ Depending on the tetrazine–dienophile pair, second-order rate constants can exceed 10^6^ M^−1^ s^−1^, making IEDDA substantially faster than CuAAC, SPAAC, and most other bioorthogonal ligation reactions.^92,93,113–116^ The irreversible extrusion of molecular nitrogen (N_2_) provides a strong thermodynamic driving force, while the excellent orthogonality of tetrazines toward naturally occurring biomolecules enables highly selective labeling in complex biological systems.^7,92,93,113,115,116^ Moreover, the reaction rates can be readily tuned through structural modification of both the tetrazine and the dienophile, and fluorogenic tetrazine probes have enabled highly sensitive imaging and detection by generating fluorescence only upon cycloaddition.^92,113,114,117,118^

In spite of its many advantages, IEDDA cycloaddition also presents several limitations. The reaction generally requires highly strained or electronically activated dienophiles to achieve its exceptional reaction rates, while the synthesis of reactive tetrazines and strained dienophiles, particularly *trans*-cyclooctene derivatives, can be synthetically demanding and relatively expensive.^7,92,93,113,115,116^ In addition, tetrazine stability represents an important practical consideration for biological applications. Tetrazine reactivity is often accompanied by a trade-off with stability, with highly reactive tetrazines being more susceptible to degradation under aqueous and biologically relevant conditions. Their stability can also be influenced by the substitution pattern and the presence of reducing or nucleophilic components such as thiols. Consequently, tetrazine design requires a balance between sufficiently rapid IEDDA kinetics and adequate stability during storage, handling, and biological exposure. More stable tetrazine and tetrazine-like systems have therefore been developed through structural and electronic modification to improve their suitability for biological applications. Another practical limitation is the configurational stability of TCO derivatives. Although TCOs exhibit exceptionally high reactivity toward tetrazines, the reactive *trans* isomer can isomerize to the thermodynamically more stable *cis* isomer. Because the *cis* isomer is considerably less reactive toward tetrazines, such isomerization can reduce the effective concentration of the bioorthogonal reaction partner and consequently diminish labeling efficiency. The development of conformationally stabilized TCO derivatives therefore represents an important strategy for maintaining high IEDDA reactivity during biological applications.^92,93,113–116,119,120^ At high reagent concentrations, limited off-target reactions with endogenous nucleophiles or unsaturated biomolecules may occur, although such effects are generally minimal under typical bioorthogonal labeling conditions.^113–115,117^ Furthermore, careful optimization of tetrazine and dienophile reactivity is often required to balance reagent stability with reaction kinetics, and the extremely rapid reaction rates can complicate stoichiometric control in multicomponent conjugation systems.^92,113–117^

Because of its ultra-fast reaction kinetics and superior bioorthogonality, IEDDA chemistry stands as a premier ligation tool across chemical biology and biomedicine. Its broad utility spans bioconjugation, protein and antibody functionalization, live-cell and *in vivo* fluorescence imaging, pretargeted PET imaging and radiotherapy, targeted drug delivery, bioorthogonal prodrug activation (decaging), hydrogel cross-linking, tissue engineering, and the design of advanced, stimuli-responsive biomaterials and polymer networks.^7,92,93,111,113–117,121^ More recently, IEDDA ligation has been extensively integrated into nanomedicine, controlled drug-release platforms, protein engineering, precision biomolecular labeling, and smart biomaterials that respond selectively to bioorthogonal cycloaddition events.^111,115,116^ Compared with classical DA cycloadditions, IEDDA combines ultrafast reaction kinetics with catalyst-free operation and complete bioorthogonality under physiological conditions, making it the benchmark bioorthogonal ligation for modern chemical biology and biomedical applications.^7,92,93,111,113–115,117^ The key differences between DA and IEDDA are summarized in Table 2.

**Table 2:** Comparative overview of DA and IEDDA click chemistries

Feature	Classical DA	IEDDA
Reactants	Electron-rich diene + electron-poor dienophile^63–66^	Electron-poor diene (typically tetrazine) + electron-rich or strained alkene/alkyne^110,111^
Reaction mechanism	Thermal [4 + 2] cycloaddition^66^	[4 + 2] cycloaddition followed by N_2_ extrusion^110,111^
Reaction conditions	Often requires heating^63,64,67,68^	Usually extremely fast, often at room temperature^92,93,113–116^
Major applications	Used mainly in polymer/material chemistry^63,64,67,68^	Widely used for bioorthogonal chemistry and bioconjugation^92,93,113,115,116^
Reversibility	May be reversible (*e.g.*, furan–maleimide)^63,64,67,68^	Essentially irreversible due to nitrogen release^92,93,113,115,116^

#### 2.2.3. SPANC

SPANC is a copper-free bioorthogonal click reaction that proceeds through a concerted [3 + 2] 1,3-dipolar cycloaddition between a nitrone and a strained cycloalkyne, typically a cyclooctyne derivative such as BCN, DBCO, DIBO, or BARAC, under mild physiological conditions without the need for transition-metal catalysts (Fig. 14). First introduced by van Delft and co-workers as an extension of strain-promoted cycloaddition chemistry, SPANC exploits the substantial ring strain of cyclooctynes to lower the activation barrier and promote rapid cycloaddition with nitrones, affording stable N-alkylated isoxazoline adducts with excellent atom economy and essentially no by-product formation.^9,34,56,104,122,123^ Mechanistically, the reaction proceeds through a thermally allowed, nearly synchronous, nonpolar 1,3-dipolar cycloaddition in which the nitrone acts as the 1,3-dipole and the strained cycloalkyne serves as the dipolarophile. The reaction kinetics can be further tuned through stereoelectronic modification of both the nitrone (*e.g.*, α-substituents or endocyclic *versus* exocyclic nitrones) and the cyclooctyne structure, with second-order rate constants ranging from approximately 1 to 60 M^−1^ s^−1^ depending on the reactants employed, which are often one to two orders of magnitude faster than those observed for conventional SPAAC reactions.^9,34,56,90,104,124,125^

**Fig. 14:**
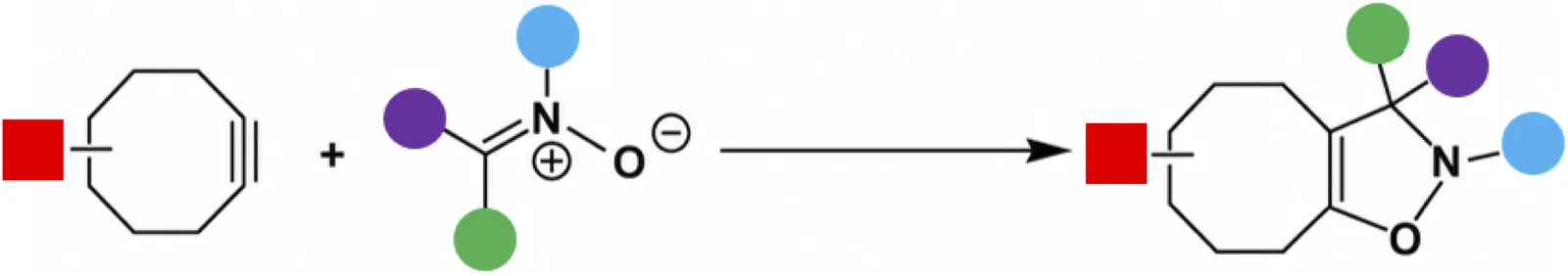
Schematic illustration of SPANC.

Relying on copper-free execution, rapid kinetics, high chemoselectivity, and broad functional-group tolerance, SPANC has gained widespread attention for its seamless performance in aqueous and physiological settings. Compared with SPAAC, SPANC generally exhibits faster reaction kinetics because nitrones are more electron-rich 1,3-dipoles than azides, promoting more efficient interaction with strained cyclooctynes and enabling rapid ligation even at relatively low reagent concentrations.^9,34,56,90,104,122^ The reaction proceeds efficiently under mild conditions without requiring metal catalysts or external activation and produces essentially no toxic by-products, making it highly suitable for the selective modification of proteins, peptides, nucleic acids, polymers, nanoparticles, and other biomolecules while preserving their biological activity.^9,34,56,90,122,126,127^ The broader implementation of SPANC is nevertheless constrained by several practical considerations. Nitrones are generally less chemically stable than organic azides, making them more susceptible to hydrolysis, oxidation, and decomposition during storage and handling. In addition, the synthesis of suitably substituted nitrones and highly strained cyclooctynes often requires multiple synthetic steps, increasing both the complexity and cost of reagent preparation compared with many other bioorthogonal ligation systems.^9,34,56,90,104,126^ Although SPANC generally proceeds faster than SPAAC, its reaction kinetics remain considerably slower than those of IEDDA ligation. Furthermore, highly electron-deficient nitrones may exhibit increased background reactivity or undesired side reactions, while the relatively bulky cyclooctyne reagents and isoxazoline products can reduce aqueous solubility, interfere with biomolecular recognition, or perturb the structure and function of small biomolecules and densely packed materials in certain applications.^9,34,56,90,126^

The combination of rapid catalyst-free ligation and tunable reactivity has enabled SPANC to find applications in bioconjugation, live-cell labeling, metabolic tagging, dual and multiplexed bioorthogonal conjugation, site-specific protein and peptide modification, metabolic labeling of bacterial cell walls, surface functionalization, hydrogel preparation, polymer synthesis, nanomedicine, and biomaterials engineering.^9,34,56,90,104,122,125–128^ The good aqueous stability of endocyclic nitrones and the broad structural diversity of both nitrones and strained cyclooctynes allow the reaction kinetics and physicochemical properties to be readily tailored for specific biological and materials applications, further expanding the versatility of SPANC. In addition, SPANC has been successfully employed for the preparation of polymer–biomolecule conjugates, functional nanomaterials, responsive polymer networks, and advanced molecular probes for imaging, molecular tracking, and controlled assembly under mild physiological conditions. Despite its current limitations, SPANC remains an important member of the bioorthogonal click chemistry toolbox, particularly for applications requiring rapid, catalyst-free ligation under physiologically compatible conditions. Compared with SPAAC, SPANC generally offers faster reaction kinetics while maintaining catalyst-free operation and excellent bioorthogonality, although the lower stability and more demanding synthesis of nitrone reagents currently limit its broader adoption relative to SPAAC and IEDDA.^9,34,56,90,104,126^

Taken together, SPAAC, IEDDA, and SPANC illustrate the progressive development of catalyst-free bioorthogonal chemistry toward increasingly rapid, selective, and tunable ligation strategies. While SPAAC established strain-promoted azide–alkyne cycloaddition as a broadly applicable platform for biological conjugation, IEDDA introduced exceptionally rapid tetrazine-based ligation, and SPANC expanded the range of bioorthogonal 1,3-dipolar cycloadditions through tunable nitrone–alkyne reactivity. These advances have substantially broadened the chemical space available for selective bond formation in living systems and provided the foundation for the development of next-generation click reactions beyond conventional bioorthogonal ligation.

### 2.3. Third generation: next-generation click chemistry

In the context of this review, third-generation click chemistry, also referred to as next-generation click chemistry, is defined as a conceptual category of emerging and advanced click platforms that introduce functional capabilities beyond the core requirements of classical and bioorthogonal click chemistry. This classification is based on dominant design principles rather than strict chronology. First-generation click chemistry primarily emphasizes efficient, robust, modular, and operationally simple bond formation for synthetic and materials applications, whereas second-generation click chemistry extends these principles to bioorthogonal transformations that must operate selectively and rapidly under physiological conditions and in the presence of complex biological functionality.^1,87,88,93^ Third-generation platforms build upon these foundations by introducing additional levels of control or structural programmability, including external stimulation, spatiotemporal control, tunable reactivity, orthogonality, multidimensional connectivity, stimulus-responsive bond formation, and specialized functionality for complex biological and materials environments.^129,130^ Thus, the distinction between the second and third generations is not absolute; rather, it reflects the dominant design objectives and functional capabilities of individual reaction platforms.

These design principles distinguish third-generation click chemistry from earlier platforms and encompass emerging and advanced click reactions developed to address increasingly demanding applications, including ultralow reactant concentrations, complex biological systems, spatiotemporally controlled transformations, and advanced materials fabrication.^7,8,65,92,129,130^ A major direction within this field is the development of highly selective, catalyst-free, and externally controllable reactions, including photoclick reactions and electrochemically activated click transformations, which provide temporal and spatial control over bond formation and have enabled applications in tissue engineering, smart biomaterials, live-cell imaging, and controlled drug delivery.^7,8,39,92^

Photoclick chemistry is particularly relevant to next-generation click chemistry because light enables external and spatiotemporal control over reaction initiation. Such systems have been applied to biomolecular conjugation, live-cell labeling, tissue engineering, hydrogel formation, and spatially patterned materials. However, their application can be limited by light penetration, phototoxicity, irradiation conditions, and the need for photocatalysts or photoinitiators in some systems.^131,132^ Electrochemical click chemistry provides another means of external control by using an applied potential to generate or modulate reactive species. This approach can provide temporal control over bond formation, although its implementation depends on electrode composition, applied potential, electrolyte, solvent, and the electrochemical stability of the reaction partners.^133–135^

In parallel, SuFEx chemistry has emerged as a powerful platform for constructing robust sulfur(vi)-containing linkages, functional polymers, molecular architectures, and advanced materials, thereby extending click chemistry beyond conventional cycloaddition reactions. PFEx further expands this concept by exploiting P(v)–F bonds as modular reaction handles for the controlled construction of multidimensional phosphorus-containing structures and, more recently, for applications in chemical biology and protein engineering.^65,129,130,136,137^

Additional advances include click-to-release strategies and mutually orthogonal ligation systems that employ environmentally benign reagents, aqueous media, and energy–efficient reaction conditions. Click-to-release chemistry further extends bioorthogonal chemistry by converting a selective reaction into the release of a masked drug, probe, or other functional molecule. This strategy has attracted interest in bioorthogonal drug activation and precision medicine, although reaction kinetics, precursor stability, and release efficiency remain important considerations.^114,117,118^ Mutually orthogonal click systems provide additional control by allowing multiple selective reactions to proceed in the same environment with minimal cross-reactivity. Such systems are useful for multiplexed labeling, sequential functionalization, and the construction of complex molecular architectures.^31,32^

Collectively, these developments have broadened the scope of click chemistry from efficient bond formation toward platforms that combine synthetic precision, biological compatibility, external control, and functional responsiveness, with applications spanning chemical biology, precision medicine, radiochemistry, nanotechnology, polymer science, and advanced materials engineering. Representative examples discussed in this section include SuFEx, PFEx, photoclick reactions, click-to-release chemistry, and other emerging externally controlled, orthogonal, and sustainable click transformations.^7,8,39,65,92,129,130,136,138^ The evolution of click chemistry from classical synthetic transformations to bioorthogonal and next-generation ligation strategies is summarized in Table 3.

**Table 3:** Classification of click chemistry reactions

Click chemistry generation	Key characteristics	Representative reactions
Classical click	High-yield, modular, robust synthetic reactions^1,14^	CuAAC, thiol–ene, thiol–yne, DA
Bioorthogonal	Selective reactions compatible with living systems^87–89^	SPAAC, IEDDA, SPANC, Staudinger ligation
Next-generation	Stimuli-responsive, externally controllable, highly tunable, multidimensional^7,8,65,92,129,130^	SuFEx, PFEx, photoclick, click-to-release, mutually orthogonal click, electrochemical click

#### 2.3.1. SuFEx click chemistry

SuFEx click chemistry is a next-generation click reaction introduced by Sharpless and co-workers in 2014 that exploits the unique reactivity of sulfur(vi)–fluoride bonds to construct highly stable sulfur-containing linkages under mild conditions. Unlike classical click reactions that rely primarily on cycloaddition chemistry, SuFEx proceeds through selective substitution at a high-oxidation-state sulfur(vi) center, typically using sulfur(vi)–fluoride hubs such as sulfonyl fluorides (RSO_2_F), fluorosulfates (ROSO_2_F), sulfamoyl fluorides (RNSO_2_F), and sulfuryl fluoride (SO_2_F_2_). These electrophiles undergo nucleophilic attack by oxygen-, nitrogen-, or carbon-centered nucleophiles, including silyl ethers, alcohols, phenols, and amines, followed by displacement of fluoride to form robust S–O, S–N, or S–C linkages with excellent selectivity, high atom economy, and minimal by-product formation.^8,21,130,139–142^ Mechanistically, SuFEx exploits the unusual combination of thermodynamic stability and latent kinetic reactivity of the S(vi)–F bond: although these bonds are relatively inert toward many common nucleophiles, appropriate activation by Lewis bases, bifluoride salts, silicon-based reagents, proton donors, or organic bases facilitates fluoride abstraction and promotes controlled nucleophilic substitution. This distinctive balance between stability and selective reactivity enables SuFEx transformations to proceed under mild, often metal-free conditions while minimizing undesired reactions commonly associated with less selective sulfur(vi) electrophiles (Fig. 15).^142–146^

**Fig. 15:**
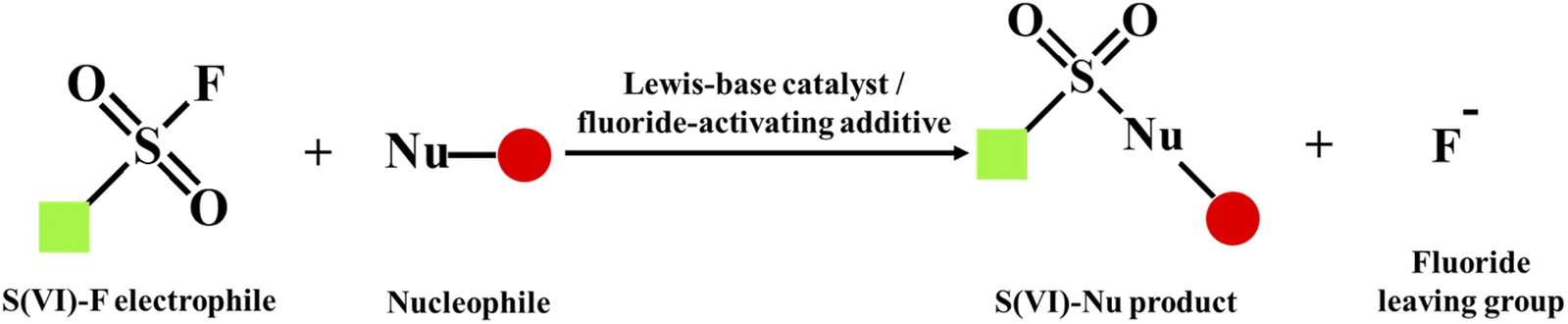
Generalized SuFEx click reaction involving nucleophilic substitution of the S(vi)–F bond by a nucleophile (Nu), with fluoride (F^−^) as the leaving group.

The particular value of SuFEx lies in its exceptional chemoselectivity, high functional-group tolerance, modularity, and the remarkable stability of the resulting sulfur(vi) linkages. Sulfur(vi)–fluoride compounds are generally stable toward water and many common functional groups under ambient conditions but can undergo rapid and selective reactions with appropriately activated nucleophiles, enabling high or near-quantitative conversions under relatively mild, often catalyst-assisted conditions. The resulting sulfonate, sulfate, sulfamide, and related sulfur(vi) linkages exhibit excellent chemical and thermal stability, while the broad range of sulfur(vi)–fluoride hubs provides considerable flexibility in molecular design. In particular, tetrahedral sulfur hubs such as sulfuryl fluoride (SO_2_F_2_) and related multifunctional sulfur(vi) reagents can serve as multidimensional connectors for constructing complex, three-dimensional molecular architectures and highly crosslinked materials. This combination of high efficiency, good compatibility with aqueous and oxygen-containing environments under appropriate conditions, structural tunability, and robust bond formation has contributed to the characterization of SuFEx as a “golden reaction” and established it as an important next-generation click chemistry platform.^8,21,130,139–141,147–149^

Despite these favorable characteristics, SuFEx chemistry also presents several limitations. Efficient sulfur(vi)–fluoride exchange may require catalytic activation using strong organic bases, silicon-based additives, or fluoride scavengers, particularly when less reactive nucleophiles or sulfur(vi)–fluoride substrates are employed. Some substrates may exhibit moisture sensitivity or reduced stability under strongly basic conditions, while poorly matched nucleophile–electrophile pairs can result in slower reaction rates, incomplete conversion, or the need for elevated temperatures and extended reaction times. In addition, the preparation of suitably functionalized sulfur(vi)–fluoride building blocks can involve multistep synthesis, increasing the complexity and cost of reagent preparation. Finally, although the exceptional stability of many SuFEx-generated sulfur(vi) linkages is a major advantage for durable materials, it can also limit subsequent bond editing, recycling, or degradation when reversibility or degradability is required.^8,21,130,139–141,143^

These limitations are particularly relevant when SuFEx chemistry is considered for biological or therapeutic applications. Although the reactivity of sulfur(vi)–fluoride electrophiles can be tuned over a broad range, their biological compatibility depends strongly on the intrinsic electrophilicity, concentration, residence time, hydrolytic stability, and local protein environment. For example, aryl fluorosulfates may exhibit limited nonspecific reactivity toward biomolecules under physiological conditions while undergoing selective covalent modification of nucleophilic amino-acid residues, including tyrosine (Tyr), lysine (Lys), and serine (Ser), when appropriately positioned within protein binding sites. However, such selectivity is context-dependent and does not necessarily imply universal biological compatibility. Accordingly, potential off-target reactivity, hydrolytic stability, cellular compatibility, proteome-wide selectivity, and cytotoxicity should be carefully evaluated when SuFEx reagents are considered for biological or therapeutic applications.^150–152^

Given its exceptional selectivity, robustness, and modularity, SuFEx chemistry has rapidly established itself as a cornerstone of next-generation click chemistry. Its versatility extends across medicinal chemistry, drug discovery, covalent probe development, chemical biology, polymer synthesis, post-polymerization modification, surface functionalization, and advanced materials science. Within medicinal and chemical biology specifically, SuFEx underpins high-throughput hit-to-lead screens, target-directed covalent inhibitor design, activity-based protein profiling, and the precise modification of nucleosides and nucleic acids. In materials science, SuFEx enables polymerization and network formation, the preparation of functional polymers, dendrimers, porous organic frameworks, hydrogels, and dynamic covalent materials, as well as layer-by-layer film assembly and the post-functionalization of reactive surfaces and coatings for applications such as biosensing and antibiofouling.^8,21,130,139–141,145,147,153,154^ The combination of highly selective S(vi)–F exchange with the exceptional stability of the resulting sulfur(vi) linkages makes SuFEx particularly attractive for the construction of complex molecular architectures and durable functional materials, establishing it as a powerful platform for advanced molecular and materials design.^141,143,155^

#### 2.3.2. PFEx

PFEx is an emerging next-generation click reaction that extends the fluoride-exchange concept of SuFEx to high-oxidation-state phosphorus(v) centers. PFEx involves the selective substitution of P(v)–F bonds in phosphorus fluoride electrophiles by nucleophiles such as aryl and alkyl alcohols, phenols, and amines, generating stable tetrahedral P(v)–O and P(v)–N linkages with high chemoselectivity.^136,137,156,157^ A generalized representation of the PFEx transformation is shown in Fig. 16. Mechanistically, the P(v)–F electrophile is activated by Lewis-base catalysis, with 1,5,7-triazabicyclo[4.4.0]dec-5-ene (TBD) identified as an effective catalyst; silicon-based additives such as hexamethyldisilazane (HMDS) can further enhance the exchange process by promoting fluoride capture and facilitating nucleophilic substitution at the phosphorus center.^137,156,158,159^ Importantly, multifunctional P(v)–F hubs can undergo controlled and sequential fluoride-exchange reactions, enabling the installation of different functional groups around the tetrahedral phosphorus center and providing access to multidimensional molecular architectures with precisely controlled connectivity.^136,137,156^

**Fig. 16:**
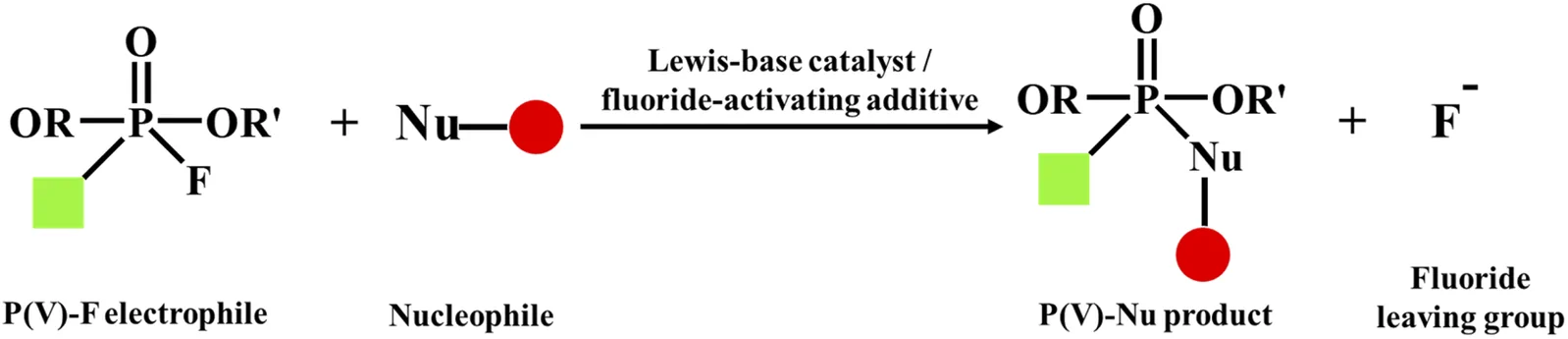
Generalized PFEx click reaction involving nucleophilic substitution of the P(v)–F bond by a nucleophile (Nu), with fluoride (F^−^) as the leaving group.

A major advantage of PFEx is its combination of high P(v)–F precursor stability with controllable latent reactivity, enabling highly chemoselective transformations under mild, metal-free conditions while maintaining good functional-group tolerance and producing stable P(v)–O and P(v)–N linkages with minimal by-product formation.^137^ The tetrahedral phosphorus center provides multiple spatially defined connection points, allowing multifunctional P(v)–F hubs to undergo controlled and sequential exchange reactions and thereby facilitating rapid access to structurally diverse three-dimensional molecules and architectures.^137,158^ Importantly, PFEx can operate orthogonally with established click reactions, including SuFEx and CuAAC, enabling sequential reaction sequences such as SuFEx–PFEx–CuAAC with minimal cross-reactivity between the different clickable functional groups.^137,159^ This combination of chemoselectivity, modularity, orthogonality, and multidimensional connectivity makes PFEx particularly attractive for diversity-oriented molecular synthesis and the rapid generation of complex functional structures.^136,137^

As a comparatively young click platform, PFEx still faces several challenges that restrict its broader implementation. Access to suitable P(v)–F building blocks can be difficult because many substrates have limited commercial availability and require specialized or multistep synthesis.^137,157^ Although P(v)–F substrates are generally stable, some phosphorus fluoride reagents also require careful handling and appropriate safety precautions because of their electrophilic and potentially hazardous nature.^137^ In addition, PFEx efficiency can vary depending on the electronic and steric properties of the nucleophile and P(v)–F electrophile; poorly nucleophilic or sterically hindered partners may require higher catalyst loadings, elevated temperatures, longer reaction times, or further reaction optimization to achieve complete conversion. The requirement for Lewis-base catalysts and, in many small-molecule applications, silicon-based activating additives can further increase the operational complexity of the reaction.^137,157^ Finally, the substrate scope, long-term stability, and compatibility of PFEx reagents with complex biological environments remain areas requiring further investigation.^136^ These considerations are particularly important for biological and therapeutic applications of PFEx. PFEx has recently expanded from small-molecule synthesis into chemical biology, with phosphoramidofluoridate-containing amino acids enabling proximity-driven covalent modification of proteins *in vitro* and in living cells. These studies demonstrate that PFEx can operate under biologically relevant conditions and can target nucleophilic residues such as histidine (His), cysteine (Cys), Tyr, and Lys. However, the biological scope and safety profile of PFEx remain less extensively characterized than those of established bioorthogonal reactions. In particular, comprehensive studies addressing proteome-wide selectivity, nonspecific protein modification, long-term cellular effects, and *in vivo* toxicity are still needed. This consideration is especially relevant because some organophosphorus(v) fluoride compounds are associated with significant biological toxicity. Therefore, the apparent stability and chemoselectivity of individual PFEx reagents should not be taken as evidence of universal biocompatibility, and systematic evaluation of cytotoxicity, hydrolytic stability, residue selectivity, and off-target protein modification will be important for future biological and therapeutic applications.^136,160,161^

PFEx has demonstrated promising applications in diversity-oriented synthesis, late-stage functionalization of drug fragments, phosphoramidate and phosphate-mimetic synthesis, covalent protein engineering, biomolecular crosslinking, and the construction of multidimensional phosphorus-rich architectures.^137,158,160^ The development of genetically encoded phosphoramidofluoridate amino acids further expands the scope of PFEx toward chemical biology and biomedical applications, including site-selective protein modification and the construction of functional biomolecular architectures.^136^ The key differences between SuFEx and PFEx in terms of their reactive centers, nucleophile scope, linkages formed, reaction kinetics, typical outcomes, and major applications are summarized in Table 4.

**Table 4:** Comparative overview of SuFEx and PFEx click chemistries

Feature	SuFEx	PFEx
Reactive center	S(vi)–F^142–145^	P(v)–F^136,137,156,157^
Typical nucleophiles	Alcohols, phenols, amines, silyl derivatives^8,21,130,139–142^	Alcohols, phenols, amines^136,137,156,157^
Primary linkages formed	S–O, S–N, S–C^8,21,130,139–142^	P–O, P–N^136,137,156,157^
Typical reaction time	Minutes to tens of minutes^148^	Tens of minutes to a few hours^137^
Typical outcome	Rapid formation of robust sulfur(vi) linkages^8,21,130,139–141,147^	Controlled P–O/P–N linkage formation and sequential exchange at multifunctional P(v) hubs^137,158^
Major applications	Materials, polymers, medicinal chemistry, chemical biology, surface functionalization^8,21,130,139–141,145,147,153,154^	Diversity-oriented synthesis, drug-fragment functionalization, protein engineering, biomolecular applications, 3D molecular architectures^136,137,158,160^

Together, SuFEx and PFEx illustrate how next-generation click chemistry has expanded beyond conventional cycloaddition reactions toward highly programmable heteroatom-centered bond-forming processes. SuFEx provides a mature and broadly applicable platform for robust sulfur(vi) linkage formation across chemical biology, medicinal chemistry, polymers, and advanced materials, whereas PFEx introduces additional structural programmability through sequential exchange at tetrahedral phosphorus centers and is beginning to extend fluoride-exchange chemistry into protein engineering and chemical biology. These developments highlight the increasing emphasis of third-generation click chemistry on modularity, orthogonality, multidimensional connectivity, and precise control over molecular and materials architecture.

## 3 Conclusions

Click chemistry has evolved from a concept focused on efficient and reliable bond formation into a diverse toolbox addressing increasingly complex synthetic, biological, and materials-related challenges. Classical click reactions established the principles of high efficiency, selectivity, modularity, and operational simplicity, while bioorthogonal chemistry extended these principles to selective transformations in living systems. Reactions such as CuAAC, SPAAC, SPANC, and IEDDA have consequently become valuable tools for molecular synthesis, biomolecular labeling, imaging, and chemical biology. The emergence of next-generation click chemistry has further expanded this toolbox. SuFEx and PFEx extend click chemistry beyond conventional cycloaddition reactions toward highly stable and structurally programmable sulfur- and phosphorus-containing architectures. Despite these advances, challenges remain in reagent stability, precursor accessibility, reaction kinetics, toxicity, and compatibility with complex biological environments. Future developments will therefore require increasingly selective, biocompatible, sustainable, and orthogonal reaction systems with improved control over kinetics and reversibility. Overall, the progression from classical to bioorthogonal and next-generation click chemistry demonstrates the increasing integration of synthetic efficiency, biological compatibility, and functional control, broadening its applications across chemical biology, precision medicine, nanotechnology, polymer science, and advanced materials engineering.

## Author contributions

Seyed Mohammad Mahdi Dadfar: conceptualization, investigation, visualization, writing – original draft, writing – review & editing. Seyed Mohammadali Dadfar: investigation, writing – review & editing.

## Conflicts of interest

The authors declare that they have no known competing financial interests or personal relationships that could have appeared to influence the work reported in this paper.

## Data Availability

No new data were created or analyzed in this study. Data sharing is not applicable to this article as all analyzed information is derived from published literature cited in the text.
